# Transcriptional downregulation of microRNA-19a by ROS production and NF-κB deactivation governs resistance to oxidative stress-initiated apoptosis

**DOI:** 10.18632/oncotarget.20235

**Published:** 2017-08-12

**Authors:** Jun Hong, Ying Wang, Bang-Chuan Hu, Liang Xu, Jing-Quan Liu, Min-Hua Chen, Jin-Zhu Wang, Fang Han, Yang Zheng, Xu Chen, Qian Li, Xiang-Hong Yang, Ren-Hua Sun, Shi-Jing Mo

**Affiliations:** ^1^ Department of Critical Care Medicine, Zhejiang Provincial People’s Hospital, People’s Hospital of Hangzhou Medical College, Hangzhou 310000, China; ^2^ Department of Neurosurgery, Huashan Hospital, Fudan University, Shanghai 200040, China

**Keywords:** microRNA-19a, ROS, NF-κB, oxidative stress, apoptosis

## Abstract

Cell apoptosis is one of the main pathological alterations during oxidative stress (OS) injury. Previously, we corroborated that nuclear factor-κB (NF-κB) transactivation confers apoptosis resistance against OS in mammalian cells, yet the underlying mechanisms remain enigmatic. Here we report that microRNA-19a (miR-19a) transcriptionally regulated by reactive oxygen species (ROS) production and NF-κB deactivation prevents OS-initiated cell apoptosis through cylindromatosis (CYLD) repression. CYLD contributes to OS-initiated cell apoptosis, for which NF-κB deactivation is essential. MiR-19a directly represses CYLD via targeting 3′ UTR of CYLD, thereby antagonizing OS-initiated apoptosis. CYLD repression by miR-19a restores the IKKβ phosphorylation, RelA disassociation from IκBα, IκBα polyubiquitination and degradation, RelA recruitment at *VEGF* gene promoter as well as VEGF secretion in the context of OS. Either pharmacological deactivation of NF-κB or genetic upregulation of CYLD compromises the apoptosis-resistant phenotypes of miR-19a. Furthermore, miR-19a is transcriptionally downregulated upon OS in two distinct processes that require ROS production and NF-κB deactivation. VEGF potentiates the ability of miR-19a to activate NF-κB and render apoptosis resistance. Our findings underscore a putative mechanism whereby CYLD repression-mediated and NF-κB transactivation-dependent miR-19a regulatory feedback loop prevents cell apoptosis in response to OS microenvironment.

## INTRODUCTION

Oxidative stress (OS), a state that reflects the balance between the systemic manifestation of reactive oxygen species (ROS) and detoxification of reactive intermediates or the resulting tissue damage, has been found in a wide range of human diseases, including Alzheimer’s disease, pulmonary edema, stroke, myocardial infarction and acute kidney injury [1-5]. Cobalt chloride (CoCl_2_) and hydrogen peroxide (H_2_O_2_) are well-characterized chemical agents widely utilized to establish OS models [6, 7]. Large amounts of ROS produced by OS trigger signal transduction and gene expression, resulting in cell apoptosis, which is the prominent contributor of functional loss and often precedes lethal insults for several years. Herein, a better understanding of molecular mechanisms concerning OS-initiated cell apoptosis could lay a framework for optimal development of innovative therapies for tissue damage.

The nuclear factor kappa enhancer binding protein (NF-κB) families, consisted of P50, P52, RelA (P65), c-Rel, and RelB, have central roles in most, if not all, physiological and pathological processes (e.g., inflammation, immunoregulation, cancer progression and cell survival). Canonical NF-κB activation cascade in response to extracellular stimuli has been comprehensively studied. In unstimulating cells, NF-κB is sequestered in the cytoplasm by IκBs, the NF-κB inhibitory proteins that belong to the proto-typical member of IκB family. Following stimulation of tumour necrosis factor-α (TNF-α) or other cytokines, proteins bearing specific ubiquitin-binding domain denoted as UBD, form complex with the Lys 63-linked polyubiquitylation of receptor interacting protein 1 (RIP1) through recruiting TNF receptor-associated factors (TRAFs) [8]. The formation of this complex subsequently facilitates the TAK1-dependent phosphorylation of IKKβ, which in turn phosphorylates IκBα at Ser32/36, leading to IκBα disassociation from NF-κB, assembly of Lys 48-linked polyubiquitylation of IκBα by SKP-CUL-F-box (SCF)-βTrCP E3 ligase, degradation of IκBα by 26S proteasome, nuclear translocation of NF-κB and transcription of NF-κB target genes [9]. Intriguingly, NF-κB transactivation is switched off through negative feedback signaling sensor cylindromatosis (CYLD), the Lys63-specific deubiquitination (DUB) enzyme that dismantles Lys63-linked poly-Ub chains from multiple NF-κB activators such as TRAF-2, TAK1 and NEMO [10]. Despite our recent work has elucidated that CYLD can serve as an apoptosis inducer under certain conditions [11], the biological functions of CYLD in OS-initiated cell apoptosis are hitherto poorly understood.

MicroRNAs (miRNAs) are endogenous small non-coding RNAs 19-24 nt in length which binds to the complementary sequences in 3′ UTR of target messenger RNAs (mRNAs) for degradation or translational inhibition and both [12]. MiRNA-19a belongs to one member of the miR-17-92 cluster that encodes six mature miRNAs, including miR-17, miR-18, miR-19a/b, miR-20 and miR-92 [13]. Functioning as an upstream inhibitor to control the expression of transglutaminase-2 (TG-2), miR-19a enhances cell invasion and metastasis of colorectal cancer [14]. Autocrine secretion-activated interleukin-6 (IL-6) in chronic lymphocytic leukemia (CLL) cells upregulates miR-19a, which disrupts toll-like receptor-7 (TLR-7) signaling [15]. It has been reported that miR-19a-activated Akt/GSK signaling is essential for glycogen synthesis in hepatocytes [16]. In addition, hypoxia-inducible factor-1α (HIF-1α)-dependent upregulation of CXCL1 expression mediated by miR-19a suppression in apolipoprotein E knockout mice promotes atherosclerosis [17]. Repression of endothelial-derived miR-19a improves blood flow recovery of ischemic limbs and hearts in aged mice [18]. Nevertheless, the precise mechanism of how miR-19a governs cell fates upon OS remains unclear.

In this study, we investigated the distinct roles of miR-19a on cell apoptosis initiated by OS and identified the possible mechanism: restoration of CYLD repression-mediated NF-κB transactivation. We also discovered a novel NF-κB/miR-19a reciprocal feedback circuit that may provide cell protection upon OS exposure. Our data unearthed a regulatory signaling cascade for sustaining NF-κB transactivation during OS and highlighted that restoring miR-19a expression as a therapeutic strategy for preventing OS-initiated apoptosis.

## RESULTS

### CYLD confers OS-initiated apoptosis via inhibiting NF-κB transactivaion

**Figure 1:**
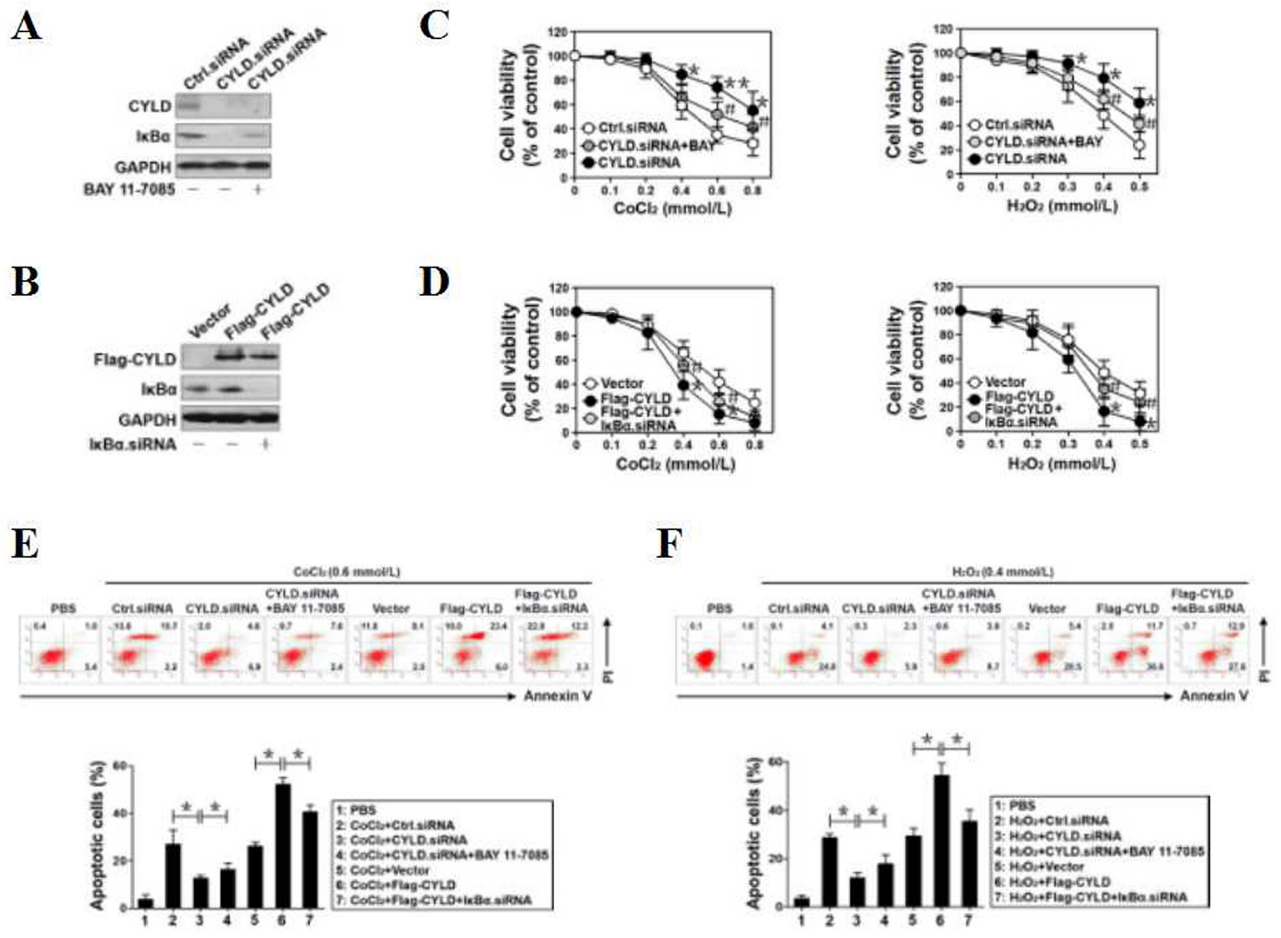
CYLD confers OS-initiated apoptosis via inhibiting NF-κB transactivation **(A)** Western blotting analysis of CYLD protein levels in PC12 cells transfected with small interfering RNA (siRNA) targeting CYLD (CYLD.siRNA) in the absence or presence of BAY 11-7085 administration. **(B)** Western blotting analysis of CYLD protein levels in PC12 cells transfected with vector expressing Flag-tagged wild-type CYLD (Flag-CYLD) in the absence or presence of IκBα.siRNA transfection. **(C)** MTT assay measuring cell viability of CYLD.siRNA-expressed PC12 cells exposed to CoCl_2_ (left panel) or H_2_O_2_ (right panel)at the indicated concentrations with or without BAY 11-7085 administration. Experiments were performed three times and data are expressed as mean ± s.d. *p < 0.05, **p < 0.01 versus Ctrl.siRNA; ^#^*p* < 0.05 versus CYLD.siRNA, one-way ANOVA, post hoc comparisons, Tukey’s test. **(D)** MTT assay measuring cell viability of Flag-CYLD-expressed PC12 cells exposed to CoCl_2_ (left panel) or H_2_O_2_ (right panel) at the indicated concentrations with or without IκBα.siRNA transfection. Experiments were performed three times and data are expressed as mean ± s.d. *p < 0.05 versus vector; ^#^*p* < 0.05 versus Flag-CYLD, one-way ANOVA, post hoc comparisons, Tukey’s test. **(E** and **F)** Representative histograms and quantification of flow cytometry with Annexin-V/PI staining in PC12 cells exposed to 0.6 mmol/L CoCl_2_ (E) or 0.4 mmol/L H_2_O_2_ (F) with CYLD.siRNA transfection in the absence or presence of BAY 11-7085 administration and with Flag-CYLD transfection in the absence or presence of IκBα.siRNA transfection, respectively. Experiments were performed three times and data are expressed as mean ± s.d. *p < 0.05, one-way ANOVA, post hoc comparisons, Tukey’s test.

CYLD switches off NF-κB signaling through its unique DUB enzyme activity [19, 20]. To further validate and extend these findings, we transfected PC12 cells with either small interfering RNA (siRNA) targeting CYLD (CYLD.siRNA) or vector encoding Flag-tagged wild-type CYLD (Flag-CYLD) and performed western-blotting to detect the levels of CYLD protein expression. In comparison with control siRNA (Ctrl.siRNA), CYLD siRNA reduced CYLD abundance, which correlates kinetically with an increased IκBα degradation (Figure 1A). Treatment with BAY 11-7085, a known NF-κB antagonist that blocks IκBα phosphorylation and NF-κB transactivation, effectively abrogated the CYLD siRNA-induced IκBα degradation (Figure 1A). In contrast, ectopic expression of Flag-CYLD, but not vector, enhanced the steady-state levels of IκBα. Nevertheless, this enhancement was substantially mitigated by IκBα siRNA (Figure 1B). Consistent with our previous study [11], the inhibitory function of CYLD on NF-κB relies on its intact catalytic activity because expression of a catalytically inactive CYLD^C601A^ mutant, in which cysteine601 is substituted by alanine, could not enhance the abundance of IκBα to the equivalent extent as wild-type CYLD did (Supplementary Figure 1A). Similar results were recapitulated in human embryonic kidney (HEK) 293T cells (Supplementary Figure 1B), suggesting that NF-κB deactivation by catalytic activity of CYLD is not restricted to cell species.

To interrogate the biological roles of CYLD on OS-initiated cell apoptosis, we treated CYLD siRNA- and Flag-CYLD-expressed PC12 cells with OS mimetic agents CoCl_2_ and H_2_O_2_ of different concentrations (from 0 to 0.8 mmol/L for CoCl_2_ and from 0 to 0.5 mmol/L for H_2_O_2_), respectively. Data from cell viability curves with MTT assay showed that either CoCl_2_ or H_2_O_2_ treatment decreased survival of PC12 cells in a dose-dependent manner, and this obviously occurred beyond concentration of 0.6 mmol/L CoCl_2_ and 0.4 mmol/L H_2_O_2_ (Figure 1C, 1D). Upon CoCl_2_ or H_2_O_2_treatment, the surviving proportion of cells bearing CYLD siRNA was much higher than that of cells bearing control siRNA. The cells bearing CYLD siRNA still had elevated survival proportion but to a lesser extent in the presence of BAY 11-7085 (*p<0.05, Figure 1C), indicating that NF-κB activation contributes to the CYLD deficiency-mediated apoptosis resistance against OS. Conversely, cells harboring wild-type CYLD were more sensitive to the CoCl_2_- or H_2_O_2_-induced apoptosis than the cells harboring vector, whereas these phenotypes were ameliorated after depleting IκBα with IκBα siRNA (*p<0.05, Figure 1D). The requirement for NF-κB for the resistant role of CYLD deficiency in OS-initiated cell apoptosis was further corroborated by the lactate dehydrogenase (LDH) release assay and flow cytometry analyses with Annexin V/PI staining, which showed that BAY 11-7085 restored the amount of LDH release (*p<0.05) and the percentage of apoptotic cells (*p<0.05) decreased by CYLD siRNA, whereas IκBα siRNA attenuated the amount of LDH release (*p<0.05) and the number of apoptotic cells (*p<0.05) increased by Flag-CYLD, upon either CoCl_2_ (Supplementary Figure 1C and Figure 1E) or H_2_O_2_ (Supplementary Figure 1D and Figure 1F) treatment, respectively. These data together suggest that CYLD confers OS-initiated apoptosis through inhibition of NF-κB transactivation.

### MiR-19a directly targets CYLD and antagonizes OS-initiated apoptosis

**Figure 2:**
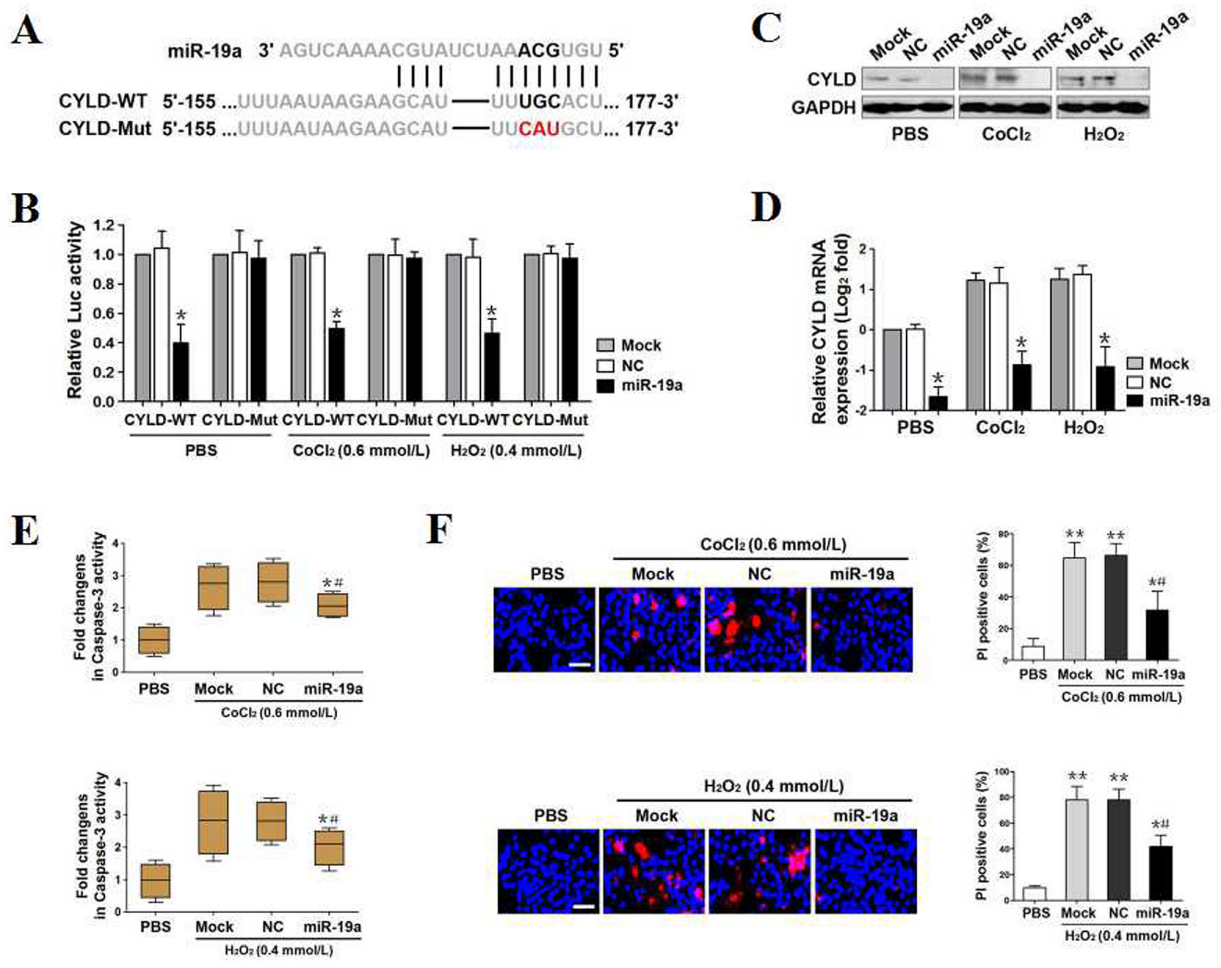
MiR-19a directly targets CYLD and antagonizes OS-initiated apoptosis **(A)** Predicted miR-19a target sequence in wild-type CYLD-3′UTR (CYLD-WT) and mutant CYLD-3′UTR containing mutated nucleotides in the miR-19a seed-pairing region (CYLD-Mut). **(B)** Luciferase assays of CYLD-3′UTR activity in PC12 cells cotransfected with WT or Mut CYLD-3′UTR reporter constructs and mock (Mock), negative control (NC) as well as miR-19a mimics (miR-19a) in the presence of PBS, 0.6 mmol/L CoCl_2_ or 0.4 mmol/L H_2_O_2_ treatment, respectively. Experiments were performed three times and data are expressed as mean ± s.d. *p < 0.05 versus Mock and NC, one-way ANOVA, post hoc comparisons, Tukey’s test. **(C)** Western-blotting examining abundance of CYLD protein expression in PC12 cells transfected with mock (Mock), negative control (NC) and miR-19a mimics (miR-19a) in the presence of PBS, 0.6 mmol/L CoCl_2_ or 0.4 mmol/L H_2_O_2_ treatment, respectively. GAPDH was used as internal control of cytoplasmic extractions. **(D)** RT-qPCR analysing levels of CYLD mRNA expression in PC12 cells transfected with mock (Mock), negative control (NC) and miR-19a mimics (miR-19a) in the presence of PBS, 0.6 mmol/L CoCl_2_ or 0.4 mmol/L H_2_O_2_ treatment, respectively. Experiments were performed five times, each with quantitative RT-PCR in technical duplicate and real-time values were normalized to glyceraldehyde 3-phosphate dehydrogenase (GAPDH). Data are expressed as mean ± s.d. *p < 0.05 versus Mock and NC, one-way ANOVA, post hoc comparisons, Tukey’s test. **(E)** Caspase-3 activity assays of PC12 cells exposed to 0.6 mmol/L CoCl_2_ (top panel) or 0.4 mmol/L H_2_O_2_ (bottom panel) with mock (Mock), negative control (NC) and miR-19a mimics (miR-19a) transfection, respectively. Experiments were performed three times and data are expressed as mean ± s.d. *p < 0.05 versus PBS; ^#^*p* < 0.05 versus Mock and NC, one-way ANOVA, post hoc comparisons, Tukey’s test. **(F)** Representative pictures (left panel) and quantification (right panel) from Hoechst and PI double-staining assay of PC12 cells exposed to 0.6 mmol/L CoCl_2_ (top panel) or 0.4 mmol/L H_2_O_2_ (bottom panel) with mock (Mock), negative control (NC) and miR-19a mimics (miR-19a) transfection, respectively. Data are expressed as mean ± s.d. *p < 0.05, **p < 0.01 versus PBS; ^#^*p* < 0.05 versus Mock and NC, one-way ANOVA, post hoc comparisons, Tukey’s test.

Both CYLD and microRNAs have crucial roles in regulating cell death [21, 22]. However, the link between these two molecules in OS-initiated apoptosis is largely unknown. Using publicly available algorithms (TargetScan), we predicted CYLD as a direct downstream target of miR-19a that contains a conserved binding site of 3′ UTR in CYLD (Figure 2A). To determine the influence of miR-19a binding on the CYLD 3′ UTR activity during OS, we transiently transfected a luciferase reporter containing the CYLD 3′ UTR with wild-type or mutant miR-19a seed-pairing region into mock-, negative control (NC)- or miR-19a-expressed PC12 cells and treated them with PBS, 0.6 mmol/L CoCl_2_ and 0.4 mmol/L H_2_O_2_, respectively. As shown in Figure 2B, miR-19a, rather than mock or NC, markedly decreased the activities of luciferase reporter fused to wild-type, but not mutant CYLD 3′ UTR in all cells tested (*p<0.05). In line with this finding, miR-19a led to a pronounced reduction in CYLD protein and mRNA expression compared with mock and NC in the western-blotting (Figure 2C) and real-time quantitative reverse transcriptase-polymerase chain reaction (RT-qPCR) analyses (*p<0.05, Figure 2D), although the abundance of CYLD protein and mRNA were eventually increased by CoCl_2_ or H_2_O_2_ single treatment. Collectively, these results not only identify CYLD as a direct downstream target of miR-19a, but also recognize a posttranslational mechanism underlying CYLD repression by miR-19a during OS.

The ability of CYLD to confer apoptosis under OS and the repression of CYLD by miR-19a prompted us to investigate the functional role of miR-19a in OS-initiated cell apoptosis. For this purpose, we separately treated mock-, NC- and miR-19a-expressed PC12 cells with 0.6 mmol/L CoCl_2_ or 0.4 mmol/L H_2_O_2_ for 24h and carried out caspase-3 activity assay to measure the cell apoptosis. In contrary to PBS, CoCl_2_ and H_2_O_2_ administration greatly elevated caspase-3 activity of mock- or NC-expressed cells but not that of miR-19a-expressed cells (*p<0.05, Figure 2E). These data were coincide with the results from LDH release assay and flow cytometry analyses with Annexin V/PI staining showing that the miR-19a-expressed cells had reduced magnitude of LDH release and percentage of apoptotic cells upon CoCl_2_ (*p<0.05) or H_2_O_2_ (*p<0.05)stimulation in contrast to the mock- or NC-expressed cells (Supplementary Figure 2). Hoechst/PI double staining also revealed that the mock- or NC-expressed cells stimulating with CoCl_2_ or H_2_O_2_, unlike the parental cells that were stimulated with PBS, had significantly increased number of PI-positive apoptotic cells (**p<0.01), whereas the miR-19a-expressed cells stimulating with CoCl_2_ or H_2_O_2_ displayed diminished number of PI-positive apoptotic cells (*p<0.05, Figure 2F). Herein, miR-19a directly targets CYLD and antagonizes OS-initiated cell apoptosis.

### CYLD repression contributes to NF-κB transactivation during OS in a miR-19a-dependent manner

**Figure 3:**
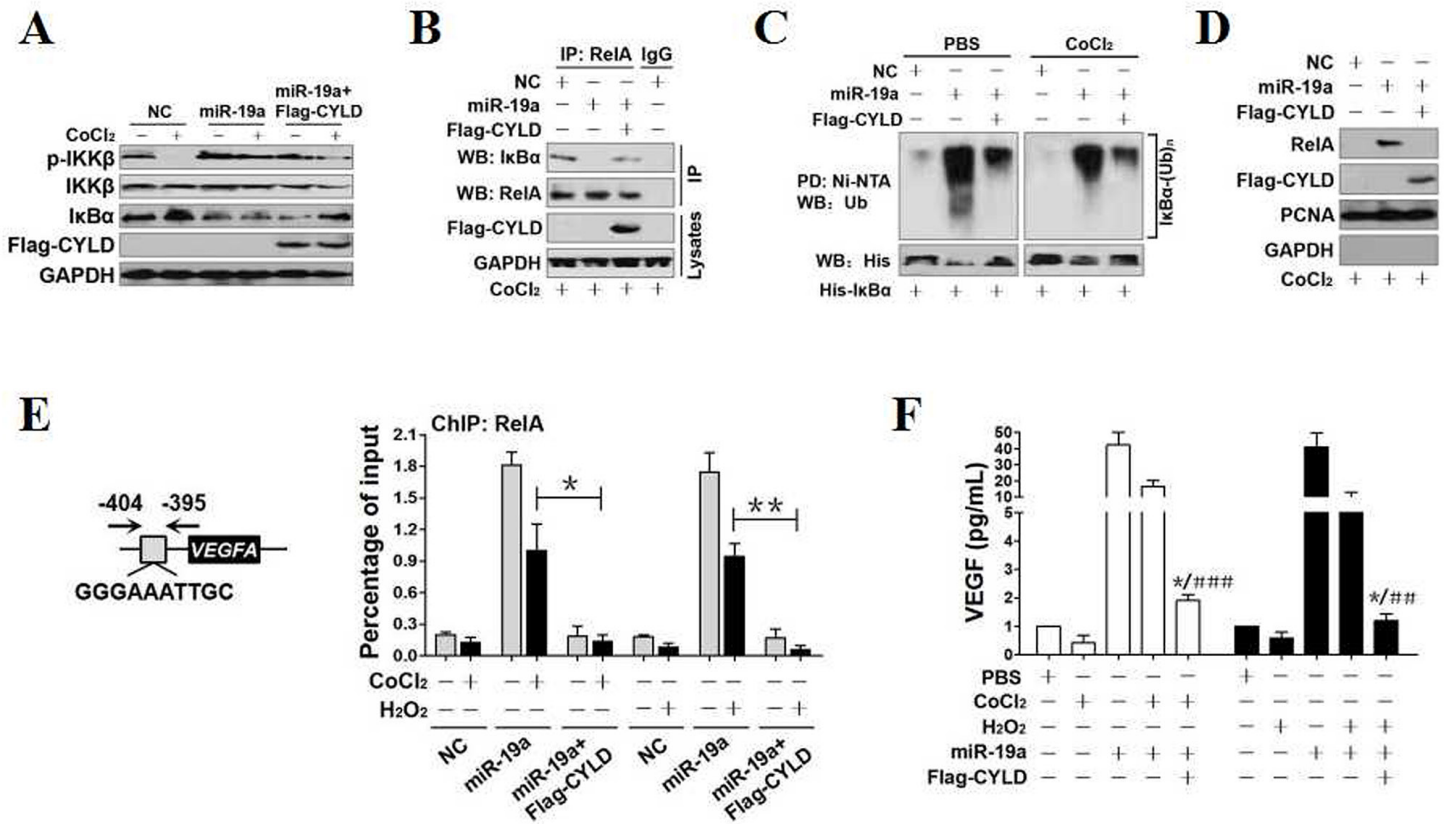
CYLD repression contributes to NF-κB transactivation during OS in a miR-19a-dependent manner **(A)** Western-blotting analyses comparing the levels of IKKβ phosphorylation and total IκBα expression in PC12 cells treated with 0.6 mmol/L CoCl_2_ in the presence of miR-19a mimics transfection and miR-19a mimics plus Flag-tagged wild-type CYLD cotransfection, respectively. **(B)** Coimmunoprecipitation assays examining the interaction between RelA and IκBα in PC12 cells treated with 0.6 mmol/L CoCl_2_ in the presence of miR-19a mimics transfection and miR-19a mimics plus Flag-tagged wild-type CYLD cotransfection, respectively. IP, immunoprecipitation; WB, western-blotting. **(C)** Cellular ubiquitination assays comparing the poly-Ub levels of IκBα in PC12 cells treated with 0.6 mmol/L CoCl_2_ in the presence of miR-19a mimics transfection and miR-19a mimics plus Flag-tagged wild-type CYLD cotransfection, respectively. PD, pull-down. **(D)** Western-blotting analyses detecting the levels of nuclear RelA accumulation in PC12 cells treated with 0.6 mmol/L CoCl_2_ in the presence of miR-19a mimics transfection and miR-19a mimics plus Flag-tagged wild-type CYLD cotransfection, respectively. **(E)** ChIP analysis for RelA binding to *VEGFA* gene promoter in PC12 cells exposed to 0.6 mmol/L CoCl_2_ and 0.4 mmol/L H_2_O_2_ in the presence of miR-19a mimics transfection and miR-19a mimics plus Flag-tagged wild-type CYLD cotransfection, respectively. Enrichment of promoter region was normalized by input and data are expressed as mean ± s.d. of at least three experiments. *p < 0.05; **p < 0.01, one-way ANOVA, post hoc comparisons, Tukey’s test. **(F)** ELISA assay for VEGF release from PC12 cell cultures exposed to 0.6 mmol/L CoCl_2_ and 0.4 mmol/L H_2_O_2_ in the presence of miR-19a mimics transfection and miR-19a mimics plus Flag-tagged wild-type CYLD cotransfection, respectively. Enrichment of promoter region was normalized by input and data are expressed as mean ± s.d. of at least three experiments. *p < 0.05 versus PBS; ***p < 0.001 versus CoCl_2_ or H_2_O_2_ plus miR-19a, one-way ANOVA, post hoc comparisons, Tukey’s test.

Since miR-19a targetedly represses CYLD which acts as the bona fide DUB of NF-κB signaling (Figure 2), we sought to determine the possible involvement of CYLD, in conjunction with miR-19a, for NF-κB transactivation upon OS exposure. To this end, we treated NC-expressed, miR-19a-expressed or miR-19a plus Flag-CYLD-coexpressed PC12 cells with 0.6 mmol/L CoCl_2_ and 0.4 mmol/L H_2_O_2_ for 24h, respectively. Western-blotting showed that IKKβ phosphorylation was almost completely abolished, while IκBα levels were dramatically enhanced, in NC-expressed cells stimulating with CoCl_2_ (Figure 3A) or H_2_O_2_ (Supplementary Figure 3A), yet miR-19a by itself potentiated the abundance of phosphorylated IKKβ but compromised the amount of IκBα. Intriguingly, miR-19a abrogated the CoCl_2_- and H_2_O_2_-induced IKKβ dephosphorylation as well as IκBα accumulation, but these phenotypes were all abrogated by reconstituted expression of Flag-CYLD in the miR-19a-replete cells (Figure 3A and Supplementary Figure 3A). In addition, western-blotting of immunoprecipitated RelA with a IκBα antibody showed that miR-19a reduced RelA binding to IκBα at endogenous levels, which could be rescued by concomitant expression of Flag-CYLD (Figure 3B and Supplementary Figure 3B). In accordance with these observations, cellular ubiquitination assay of IκBα immobilized on Ni^2+^-nitrilotriacetic acid (NTA)-sepharose beads with anti-Ub antibodies showed that CoCl_2_ and H_2_O_2_ reduced, while miR-19a augmented the poly-Ub levels of IκBα. Either CoCl_2_ or H_2_O_2_ failed to dismantle IκBα poly-ubiquitination in miR-19a-expressed cells as efficiently as did in NC-expressed cells, and even so, reconstituted expression of Flag-CYLD in miR-19a-expressed cells restored the ability of CoCl_2_ and H_2_O_2_ to dismantle IκBα poly-ubiquitination (Figure 3C and Supplementary Figure 3C). These results were further supported by fractionation analyses showing that Flag-CYLD successfully blocked nuclear RelA localization in miR-19a-expressed cells under CoCl_2_ and H_2_O_2_ stimulation (Figure 3D and Supplementary Figure 3D). Our data strongly suggest that miR-19a restores IKKβ phosphorylation upon OS via repression of CYLD, thereby resulting in disruption of IκBα:RelA complex and subsequent IκBα poly-ubiquitination as well as degradation, which in turn promotes RelA nuclear translocation.

Take into account that nuclear RelA accumulation leads to transcription of its downstream target gene VEGF [23], we thus utilized chromatin immunoprecipitation (ChIP) to detect whether the miR-19a-dependent CYLD repression affects RelA binding to the promoter of *VEGF* gene under OS. Anti-RelA antibody (Figure 3E), rather than the nonrelated immunoglobulin G (IgG) (Supplementary Figure 3E), pulled down the promoter region of *VEGFA* gene in all cells tested. Either CoCl_2_ or H_2_O_2_ administration impaired the ability of RelA binding to *VEGFA* promoter. Of note, miR-19a remarkably alleviated the inhibitory role of CoCl_2_ or H_2_O_2_ on the RelA binding to *VEGFA* promoter, the effects that can be counteracted by concurrent expression of Flag-CYLD (*p<0.05 and**p<0.01). Meanwhile, miR-19a restored the secretion of VEGF diminished by CoCl_2_ or H_2_O_2_, but this restoration could be blunted by Flag-CYLD (**p<0.01and ***p<0.001, Figure 3F). Taken together, these results implicate that miR-19a facilitates recruitment of RelA to *VEGF* gene promoter and enhances VEGF release from cells under OS, for which CYLD repression is required.

### CYLD repression and NF-κB transactivation is instrumental for the apoptosis-resistant phenotypes of miR-19a upon OS

**Figure 4:**
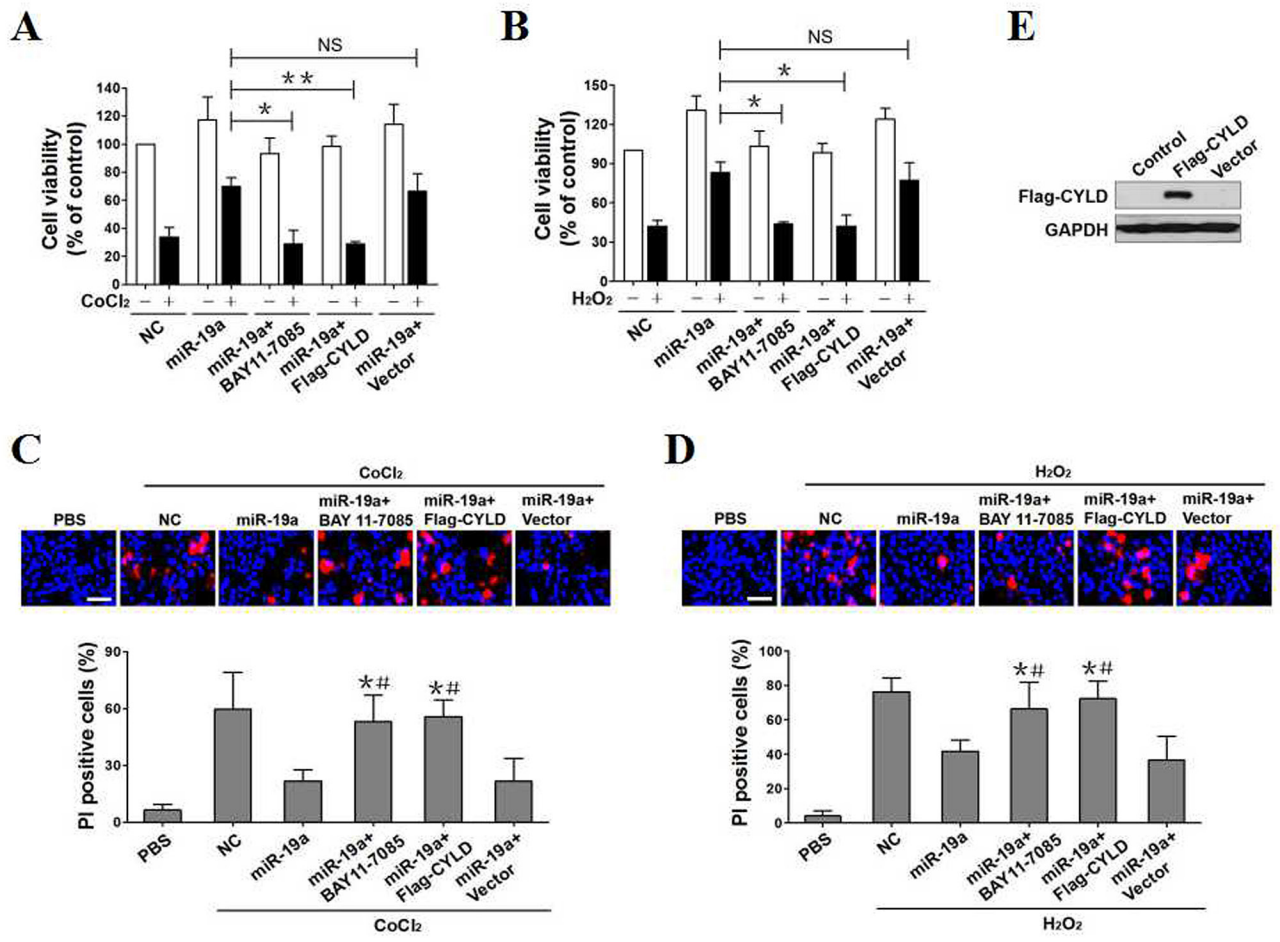
CYLD repression and NF-κB transactivation promotes the resistant phenotypes of miR-19a in OS-initiated apoptosis **(A)** PC12 cells with miR-19a mimics transfection, with miR-19a mimics transfection plus 5 μmol/L BAY 11-7085 pretreatment, with miR-19a mimics plus Flag-tagged wild-type CYLD cotransfection and with miR-19a mimics plus empty vector cotransfection were separately treated with or without 0.6 mmol/L CoCl_2_ for 24h and the cell viabilities were measured by MTT assay. Experiments were performed three times and data are expressed as mean ± s.d. *p < 0.05, **p < 0.01. NS, not significant, one-way ANOVA, post hoc comparisons, Tukey’s test. **(B)** PC12 cells with miR-19a mimics transfection, with miR-19a mimics transfection plus 5 μmol/L BAY 11-7085 pretreatment, with miR-19a mimics plus Flag-tagged wild-type CYLD cotransfection and with miR-19a mimics plus empty vector cotransfection were separately treated with or without 0.4 mmol/L H_2_O_2_ for 24h and the cell viabilities were measured by MTT assay. Experiments were performed three times and data are expressed as mean ± s.d. *p < 0.05. NS, not significant, one-way ANOVA, post hoc comparisons, Tukey’s test. **(C)** Representative pictures (top panel) and quantification (bottom panel) from Hoechst and PI double-staining assay of PC12 cells exposed to 0.6 mmol/L CoCl_2_ with miR-19a mimics transfection, with miR-19a mimics transfection plus 5 μmol/L BAY 11-7085 pretreatment, with miR-19a mimics plus Flag-tagged wild-type CYLD cotransfection and with miR-19a mimics plus empty vector cotransfection, respectively. Data are expressed as mean ± s.d. *p < 0.05 versus PBS; ^#^*p* < 0.05 versus miR-19a, one-way ANOVA, post hoc comparisons, Tukey’s test. **(D)** Representative pictures (top panel) and quantification (bottom panel) from Hoechst and PI double-staining assay of PC12 cells exposed to 0.4 mmol/L H_2_O_2_ with miR-19a mimics transfection, with miR-19a mimics transfection plus 5 μmol/L BAY 11-7085 pretreatment, with miR-19a mimics plus Flag-tagged wild-type CYLD cotransfection and with miR-19a mimics plus empty vector cotransfection, respectively. Data are expressed as mean ± s.d. *p < 0.05 versus PBS; ^#^*p* < 0.05 versus miR-19a, one-way ANOVA, post hoc comparisons, Tukey’s test. **(E)** Western-blotting assessing abundance of Flag-CYLD protein expression in PC12 cells transfected with Flag-tagged wild-type CYLD and empty vector, respectively.

To investigate whether NF-κB transactivation is the critical molecular mechanism for the apoptosis-resistant role of miR-19a under OS, we preincubated miR-19a-expressed PC12 cells with 5 μmol/L BAY 11-7085 for 2h, and then exposed them to 0.6 mmol/L CoCl_2_ and 0.4 mmol/L H_2_O_2_ for a further 24h, respectively. Compared with NC, miR-19a effectively impeded the CoCl_2_- and H_2_O_2_-reduced cell viability, but BAY 11-7085 preincubation reversed this impediment (*p<0.05, Figure 4A, 4B). Forced expression of miR-19a, rather than NC, abrogated the caspase-3 activity elevated by CoCl_2_ or H_2_O_2_, which was partially rescued after BAY 11-7085 administration (*p<0.05, Supplementary Figure 4A, 4B). Upon either CoCl_2_ or H_2_O_2_ single treatment, the number of PI-positive apoptosis staining in miR-19a-expressed cells were much less than those in NC-expressed cells, but this did not occur in the presence of BAY 11-7085 (*p<0.05, Figure 4C, 4D). These findings led us to the proposal that NF-κB transactivation is instrumental for the miR-19a-mediated apoptosis resistance against OS.

We next explored the invovlement of CYLD in the resistant action of miR-19a against OS-initiated apoptosis through stimulating miR-19a plus Flag-CYLD-coexpressed PC12 cells with CoCl_2_ or H_2_O_2_ (Figure 4E) and detecting the resultant effects. Cell viability, as measured by MTT assay, was decreased after CoCl_2_ or H_2_O_2_ administration. Introducing miR-19a expression efficiently alleviated the cell viability decreased by CoCl_2_ or H_2_O_2_, and exogenously expressing Flag-CYLD instead of empty vector successfully restored the ability of CoCl_2_ or H_2_O_2_ to induce cell death (**p<0.01 and *p<0.05, Figure 4A, 4B), revealing that miR-19a antagonizes OS-initiated cell apoptosis via repressing CYLD. The MTT data were further confirmed by the observation that the miR-19a-expressed or miR-19a plus empty vector-coexpressed cells underwent CoCl_2_ or H_2_O_2_ treatment had much lower caspase-3 activities and percentage of apoptotic cells in comparison with the NC-expressed cells, while the miR-19a plus Flag-CYLD-coexpressed cells did not (*p<0.05, Supplementary Figure 4). Notably and in agreement with these results, miR-19a diminished the number of PI-positive staining in cell cultures with CoCl_2_ or H_2_O_2_ stimulation, the effect that was robustly perturbed when simultaneous expression of Flag-CYLD (*p<0.05, Figure 4C, 4D). Our data mentioned above thus suggest that CYLD repression and NF-κB transactivation governs the resistant role of miR-19a against OS-initiated cell apoptosis.

### MiR-19a is transcriptionally downregulated in response to OS through two distinct pathways orchestrated by ROS production and NF-κB deactivation

The resistant role of miR-19a in OS-initiated cell apoptosis by NF-κB signaling raised the possibility that miR-19a might be negatively regulated by OS. To approach this, we stimulated PC12 cells with 0.6 mmol/L CoCl_2_ or 0.4 mmol/L H_2_O_2_ and assessed the change of miR-19a expression using RT-qPCR. CoCl_2_ or H_2_O_2_ stimulation progressively reduced miR-19a mRNA levels and this was partially blocked by addition of cell-permeant antioxidant N-acetylcysteine (NAC) in a time-dependent manner (*p<0.05, Supplementary Figure 5A, 5B). To evaluate whether OS transcriptionally downregulates miR-19a, we performed luciferase reporter assay to detect miR-19a promoter activity in PC12 cells expressed pGL3-miR-19a-Luc containing the full-length miR-19a promoter. As shown in Supplementary Figure 5C and 5D, the CoCl_2_- or H_2_O_2_-stimulated cell shad lower levels of miR-19a promoter activity that could be restored by NAC (*p<0.05). Because NAC quenches intracellular reactive oxygen species (ROS) generation [24], the transcriptional downregulation of miR-19a by CoCl_2_ or H_2_O_2_ is ROS-dependent.

**Figure 5:**
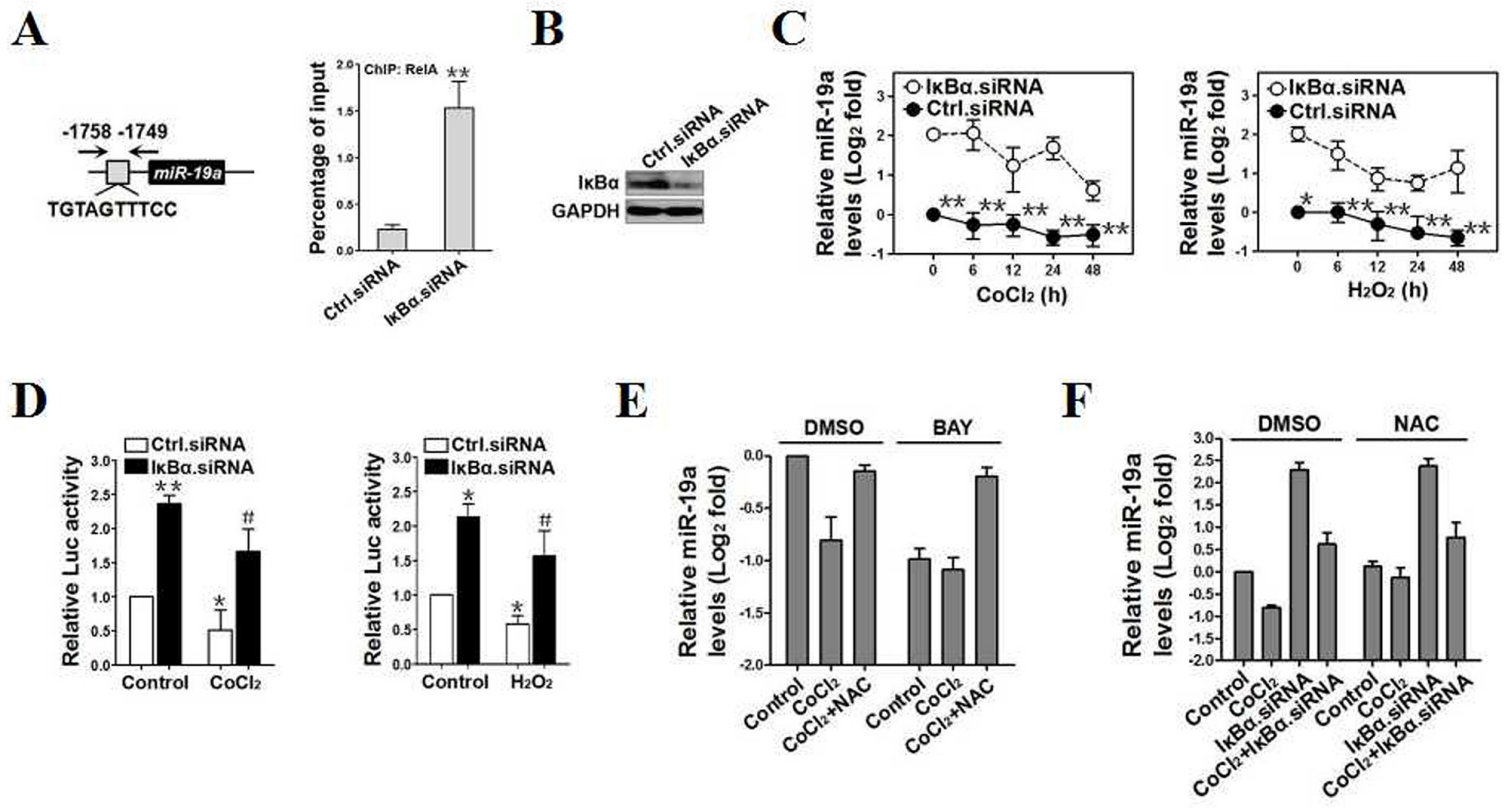
MiR-19a is transcriptionally dowregulated in response to OS through two distinct pathways orchestrated by ROS production and NF-κB deactivation **(A)** ChIP analysis for RelA binding to miR-19a promoter in PC12 cells transfected with control.siRNA (Ctrl.siRNA) and IκBα.siRNA, respectively. Enrichment of promoter region was normalized by input and data are expressed as mean ± s.d. of at least three experiments. **p < 0.01. Two-sided Student’s *t* test was used to calculate the *P* value. **(B)** Western-blotting examining abundance of IκBα protein expression in PC12 cells transfected with control.siRNA (Ctrl.siRNA) and IκBα.siRNA, respectively. **(C)** RT-qPCR comparing levels of miR-19a mRNA expression in PC12 cells treated with CoCl_2_ (left panel) or H_2_O_2_ (right panel)for the indicated times in the presence of control.siRNA (Ctrl.siRNA) or IκBα.siRNA transfection. Experiments were performed five times, each with quantitative RT-PCR in technical duplicate and real-time values were normalized to RNU6b. Data are expressed as mean ± s.d. *p < 0.05, **p < 0.01 versus IκBα.siRNA, one-way ANOVA, post hoc comparisons, Tukey’s test. **(D)** Luciferase assays of miR-19a promoter activity in PC12 cells treated with CoCl_2_ (left panel) or H_2_O_2_ (right panel)in the presence of control.siRNA (Ctrl.siRNA) or IκBα.siRNA transfection. Experiments were performed three times and data are expressed as mean ± s.d. *p < 0.05, **p < 0.01 versus Ctrl.siRNA; ^#^*p* < 0.05 versus Ctrl.siRNA plus CoCl_2_ or H_2_O_2_, one-way ANOVA, post hoc comparisons, Tukey’s test. **(E)** RT-qPCR evaluating levels of miR-19a mRNA expression in CoCl_2_-treated PC12 cells with N-acetylcysteine (NAC) treatment in the presence or absence of BAY 11-7085 administration. Experiments were performed five times, each with quantitative RT-PCR in technical duplicate and real-time values were normalized to RNU6b. Data are expressed as mean ± s.d. **(F)** RT-qPCR comparing levels of miR-19a mRNA expression in CoCl_2_-treated PC12 cells with IκBα.siRNA transfection in the presence or absence of N-acetylcysteine (NAC) treatment. Experiments were performed five times, each with quantitative RT-PCR in technical duplicate and real-time values were normalized to RNU6b. Data are expressed as mean ± s.d.

Despite our data clearly demonstrate that OS transcriptionally downregulates miR-19a by inducing ROS production, this may not be the unique mechanism because NAC only partially impairs the inhibitory role of OS in miR-19a transcription. Analysis of miR-19a promoter identified a putative NF-κB binding sequence-1758TGTAGTTTCC-1749 (Figure 5A, *left panel*), which matches appropriately with the NF-κB binding consensus sequence GGGRNNYYCC (N, any base; R, purine; and Y, pyrimidine) [25], indicating that NF-κB might be essential for the inhibition of OS on miR-19a. Indeed, ChIP assay revealed that the interaction of RelA with miR-19a promoter was dramatically enhanced after IκBα depletion using IκBα siRNA (**p<0.01, Figure 5A, 5B and Supplementary Figure 6A). The mRNA levels and promoter activity of miR-19a were augmented upon NF-κB activation by silencing IκBα with IκBα siRNA (*p*<0.01 and *p*<0.05, Figure 5C, 5D) but were reduced by deactivating NF-κB with BAY 11-7085 (*p<0.05, Supplementary Figure 6B, 6C) or RelA siRNA (*p<0.05 and **p<0.01, Supplementary Figure 6D-6F). We also noticed that NAC, which sustains transcriptional activity of miR-19a as shown in Supplementary Figure 5, abrogated the decreased mRNA levels of miR-19a by CoCl_2_ or H_2_O_2_ irrespective of BAY 11-7085 preincubation (Figure 5E and Supplementary Figure 7A), suggesting that NF-κB deactivation may not engage in the transcriptional repression of miR-19a mediated by ROS production in response to OS. Vice versa, administration of NAC did not influence the levels of miR-19a mRNA expression in IκBα-deficient cells with CoCl_2_ or H_2_O_2_ stimulation in contrast to their counterparts administrated with DMSO (Figure 5F and Supplementary Figure 7B). Together with the results of CYLD repression and NF-κB transactivation by miR-19a, our data identify a CYLD/NF-κB/miR-19a reciprocal feedback loop and implicate that miR-19a might be transcriptionally repressed upon OS via two distinct pathways regulated by ROS production and NF-κB deactivation.

### MiR-19a qualifies as a potential therapeutic target for cell survival under OS

**Figure 6:**
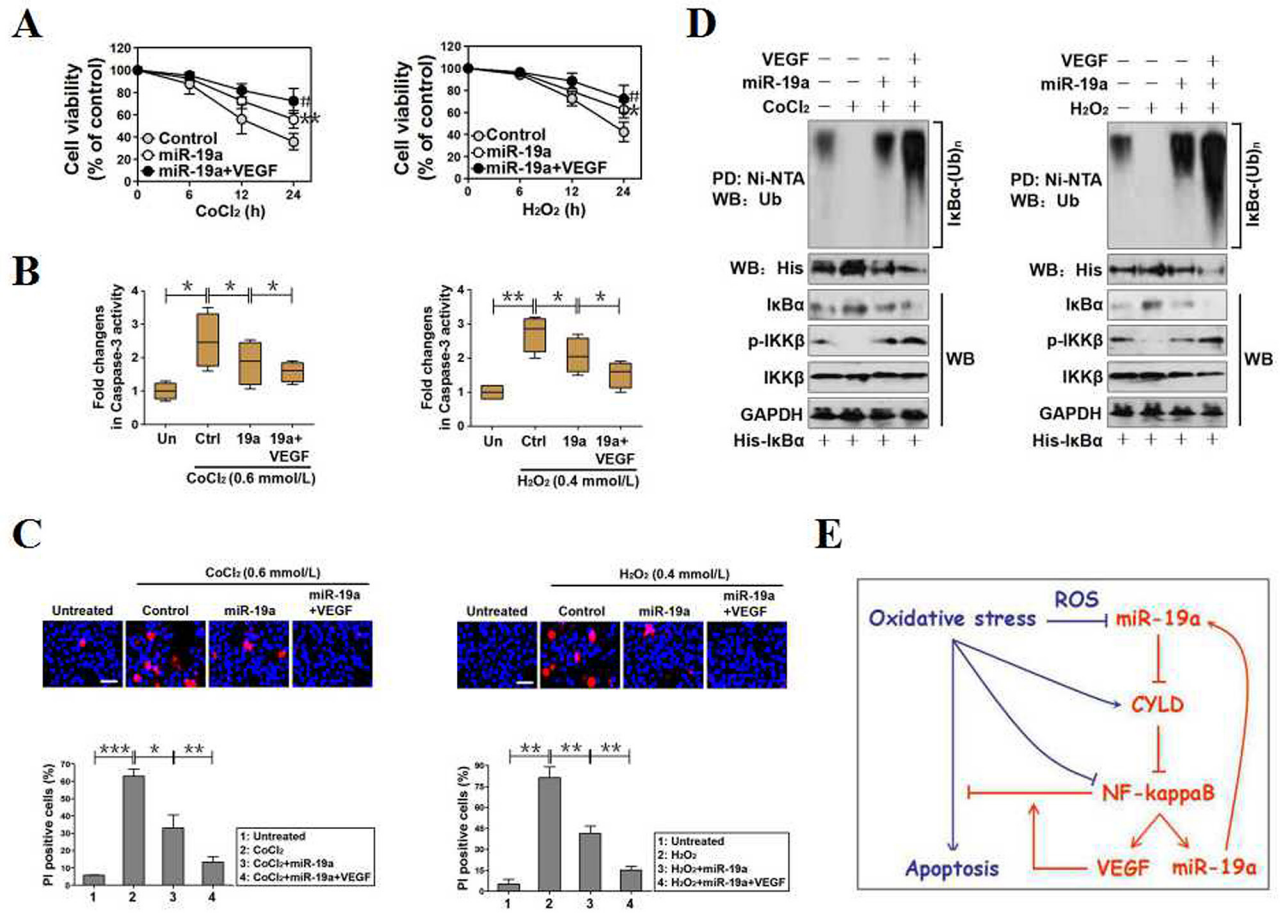
MiR-19a functions as a potential therapeutic target for cell survival under OS **(A)** PC12 cells with miR-19a mimics transfection were treated with 0.6 mmol/L CoCl_2_ (left panel) or 0.4 mmol/L H_2_O_2_ (right panel) for the indicated times in the presence or absence of 100 ng/mL VEGF pretreatment and the cell viabilities were measured by MTT assay. Experiments were performed three times and data are expressed as mean ± s.d. **p < 0.01 versus control; ^#^*p* < 0.05 versus miR-19a, one-way ANOVA, post hoc comparisons, Tukey’s test. **(B)** Caspase-3 activity assays of miR-19a-expressed PC12 cells treated with CoCl_2_ (left panel) or 0.4 mmol/L H_2_O_2_ (right panel) for 24h in the presence or absence of 100 ng/mL VEGF pretreatment. Experiments were performed three times and data are expressed as mean ± s.d. *p < 0.05, one-way ANOVA, post hoc comparisons, Tukey’s test. **(C)** Representative pictures (top panel) and quantification (bottom panel) from Hoechst and PI double-staining assay of miR-19a-expressed PC12 cells treated with CoCl_2_ (left panel) or 0.4 mmol/L H_2_O_2_(right panel) for 24h in the presence or absence of 100 ng/mL VEGF pretreatment. Data are expressed as mean ± s.d. *p < 0.05; **p < 0.01; ***p < 0.001, one-way ANOVA, post hoc comparisons, Tukey’s test. **(D)** Cellular ubiquitination assays comparing the poly-Ub levels of IκBα in miR-19a-expressed PC12 cells treated with CoCl_2_ (left panel) or 0.4 mmol/L H_2_O_2_ (right panel) in the presence or absence of 100 ng/mL VEGF pretreatment. **(E)** Proposed schematic illustrating a pivotal role for miR-19a in promoting cell survival under OS by CYLD repression-mediated and NF-κB transactivation-dependent regulatory feedback loop.

We previously demonstrated that vascular endothelial growth factor (VEGF) prevents PC12 cells from OS-initiated apoptosis via activating NF-κB [26]. Given that miR-19a exerts apoptosis-resistance under OS, we hypothesized that miR-19a might serve as a putative therapeutic target for cell survival. To examine whether VEGF would act additively with miR-19a to render apoptosis resistance against OS, we seeded miR-19a-expressed PC12 cells in 96-well dishes and exposed them to 0.6 mmol/L CoCl_2_ and 0.4 mmol/L H_2_O_2_ for 0, 6, 12 and 24 hours with or without 100 ng/mL recombinant human VEGF (rhVEGF) preincubation, respectively. Indeed, the miR-19a-replete cells with rhVEGF preincubation were less sensitive to the CoCl_2_- or H_2_O_2_-induced apoptosis than the cells without (*p<0.05, Figure 6A and Supplementary Figure 7C, 7D). The synergistic role of VEGF with miR-19a was further validated by both caspase-3 activity assay and Hoechst33342/PI staining analyses, showing that VEGF potentiated the capacity of miR-19a to reduce caspase-3 activity (*p<0.05, Figure 6B) and PI-positive apoptotic staining (**p<0.01, Figure 6B) in the presence of CoCl_2_ or H_2_O_2_. Likewisely, the steady-state levels of IκBα ubiquitination and IKKβ phosphorylation in miR-19a-replete cells with VEGF preincubation upon CoCl_2_ or H_2_O_2_ exposure were much higher than those in the cells without (Figure 6D). Our data suggest that miR-19a serves a potential therapeutic target for cell survival under OS since cytokine-derived protective agent strengthens the antiapoptotic efficacy of miR-19a when used in combination (Figure 6E).

## DISCUSSION

ROS-triggered cell death via caspase-dependent or caspase-independent fashion has been described in a large number of literature [27, 28]. Additionally, the intrinsic nature of OS with respect to lethal insults in the development and progression of tissue damage has been commented on the literature. In sharp contrast, the mechanims underlying miRNA-mediated apoptosis resistance during OS have not been comprehensively studied. Our previous work demonstrated that VEGF-dependent NF-κB transactivation antagonizes PC12 cells apoptosis induced by OS [26]. We postulated that NF-κB might function as a critical survival signal in response to extracellular cytokine stimulation that propels cells to transactivate miRNA, which would increase resistance against OS-initiated cell apoptosis.

In this study, loss-of-function and gain-of-function data show that depletion of CYLD compromises, while overexpression of CYLD potentiates PC12 cells apoptosis initiated by OS. Consistent with the notion that NF-κB signalling could be turned off through negative feedback mechanism involving CYLD-mediated ubiquitin (Ub) deconjugation [29], the CYLD depletion-compromised and CYLD overexpression-potentiated cell apoptosis are separately overrided by NF-κB inhibitor BAY 11-7085 and IκBα siRNA. Analyses using publicly available algorithms and the results of our current study identify CYLD as a direct downstream target of miR-19a. The relevance of miR-19a to apoptosis resistance under OS is demonstrated by our data showing that miR-19a-replete cells are significantly less vulnerable to the OS-initiated apoptosis than their counterparts. The functional and mechanistic data reveal that the ability of miR-19a to prevent cells against OS-initiated apoptosis is, at least part, attributed to its ability to restore NF-κB transactivation through repression of CYLD via targeting CYLD 3′ UTR for degradation. Indeed, miR-19a restores the IKKβ phosphorylation, cytoplasmic IκBα ubiquitination and degradation, disruption of IκBα:RelA complex, RelA nuclear localization and recruitment at *VEGFA* gene promoter as well as VEGF secretion in OS-stimulated cells, the effects that are all rescued by expression of wild-type CYLD. Either pharmacological deactivation of NF-κB or genetic upregulation of CYLD within miR-19a-replete cells largely abolishes the apoptosis-resistant phenotypes of miR-19a. The aforementioned results thereby suggest that the CYLD repression-dependent NF-κB transactivation is engaged in the miR-19a-mediated apoptosis resistance. In addition to discover that miR-19a is transcriptionally repressed by OS in a ROS-dependent manner, we also recognize a reciprocal regulation feedback between CYLD/NF-κB and miR-19a upon OS. Through this response, each component logically implements feedback on itself, which changes cells from a homeostatic state to an OS state. This is supported by our observations that miR-19a transcription is repressed by OS but partially mitigated when NF-κB has been activated. Following OS stimulation, transcriptional repression of miR-19a is considered to be a crucial event associated with NF-κB deactivation and occurrence of cell apoptosis. Indeed, OS blocks NF-κB transactivation, which in turn represses miR-19a transcription, disrupts the balance of CYLD/NF-κB/miR-19a regulatory feedback loop and results in cell apoptosis. Cytokines, such as nerve growth factor (NGF), epidermal growth factor (EGF) and platelet-derived growth factor (PDGF), are shown to promote cell survival through activating NF-κB [30-32]. This may explain why the apoptosis-resistant phenotypes of miR-19a against OS could be strengthened by VEGF in our current study. Our data underscore that restoring miR-19a expression might be beneficial for insults in which OS-initiated cell apoptosis is a prominent pathological hallmark. Our future work will aim at exploring the precise mechanisms of how miR-19a activates NF-κB and determining whether other signalling molecules also play important roles in the miR-19a-mediated apoptosis resistance.

There are, however, some limitations in our work. Firstly, miR-19a is estimated to target distinct genes (e.g., OTUD1, USP37 and KLF10), their functions might thus be conceivably as critical as CYLD in governing cell apoptosis during OS. Because the distinct characterization of a miRNA heavily relies on the identifiation of its targets and its influences on their post-translational modification, these targets may also be essential for the miR-19a-dependent apoptosis resistance. Meanwhile, the possibility that CYLD downregulation in response to miR-19a overexpression occurs secondarily as a consequence of these targets’ degradation could not be excluded. Secondly, it has been well-established that other DUBs, including UBXN1 and A20, are also involved in the termination of NF-κB [33, 34]. Our results show that reconsitituted expression of CYLD in miR-19a-replete cells only partially deactivates NF-κB, suggesting that CYLD might cooperate with other DUBs to switch off NF-κB signalling. Herein, whether the inhibition of other DUBs on NF-κB under OS can be rescued by miR-19a requires further investigation.

In summary, our study for the first time provide new sights into the resistant phenotype of miR-19a in OS-initiated apoptosis through its previously unappreciated role in CYLD repression-dependent NF-κB transactivation. We unearth a CYLD/NF-κB/miR-19a regulatory feedback loop that may represent a potential mechanism underlying apoptosis resistance under OS. We also demonstrate that the combination of cytokine-derived protective agent with miR-19a has a more powerful impediment on OS-initiated apoptosis than used single, implicating that miR-19a is a high-quality target for maintaining cell survival, and this may hold promise for development of novel therapeutic strategies against tissue damage driven by OS.

## CONCLUSION

In conclusion, our study is the first what we believe to report that miR-19a transcriptionally regulated by ROS production and NF-κB deactivation renders apoptosis resistance to OS through repression of DUB enzyme CYLD. The exact mechanisms of how miR-19a exerts its anti-apoptotic activity and whether other signalling molecules are also invovled in the transcriptional regulation of miR-19a still deserve to be further investigated.

## MATERIALS AND METHODS

### Cell culture and transfection

The culture protocols for PC12 cells were characterized previously [26, 35]. Briefly, cells were cultured in Dulbecco’s modified Eagle’s medium (Gibco, Carlsbad, USA) supplemented with 10% heat-inactivated horse serum, 5% fetal bovine serum (FBS), 100 U/mL penicillin and 100 mg/mL streptomycin under a humidified atmosphere at 37°C with 5% CO_2_. 293T cells were maintained in DMEM containing 10% FBS, 100 mg/mL penicillin and streptomycin and cultured at 37°C in 5% CO_2_ and 95% air. MiR-19a mimics and the negative controls of lin4 miRNA mimics were purchased from GenePharma (Shanghai, China) and transfected at a final concentration of 100 nM in the cells using HiPerFect Transfection Reagent (Qiagen, Hilden) according to the procedure described in the manufacturer’s recommendations. CYLD^C601A^ mutant was generated using the QuickChange^®^ Site-Directed Mutagenesis Kit (Agilent Technologies, Santa Clara, CA) as indicated previously [11]. For siRNA and plasmid DNA transfection, cells were transfected with specific small interfering RNA (siRNA) duplex oligonucleotides targeting CYLD (GenePharma, Shanghai, China), RelA siRNA (Santa Cruz, CA), IκBα siRNA (Santa Cruz, CA) or pReceiver-M11 vector expressing CYLD (GeneCopoeia, Rockville, USA) using Lipofectamine 2000 (Invitrogen) according to the manufacturer’s instructions as previously described [36].

### Cell viability assay

Cell viability was determined by 3-(4,5-dimethylthiazol-2-yl)-2,5-diphenyltetra- zolium bromide reduction (MTT) assay as previously described [11, 35]. In brief, the indicated cells were seeded in 96-well plates at a density of 2×10^4^ per well and underwent various treatments. Before the end of the experiment, 20 μl MTT (5 mg/ml; Sigma-Aldrich) was added and the plates were incubated at 37°C for 4 h. Subsequently, 150 mL dimethyl sulfoxide was added to dissolve formazan and the absorbance was measured at 570 nm by spectrometer (Wellscan MK3; Labsystems Dragon).

### Lactate dehydrogenase (LDH) release assay and enzyme-linked immunosorbent assay (ELISA)

LDH release assay was performed using LDH detection kits (Beyotime, China) as previously described [37]. Briefly, the indicated cells underwent various conditions were exposed to 10% Triton X-100. Samples were then collected and centrifuged at 1000 rpm at 4°C for 5 min. LDH levels in the supernatant were determined by measuring the changes in absorbance at 490 nm. For ELISA, the indicated cells were plated in 6-well plates at a density of 1×10^5^ cells/well. After the end of experiment, VEGF secreted from cells into culture supernatant was directly measured using the enzyme-linked immunosorbent assay (ELISA) kit (RayBiotech) according to manufacturer’s instructions.

### Apoptosis measurement

The apoptotic analyses of cells were measured by flow cytometry with Annexin-V/PI staining, caspase-3 activity assay and Hoechst33342-PI staining as described previously [11, 37].

### Dual-luciferase reporter assay

Luciferase reporter assays were carried out as described previously [11, 35]. The CYLD 3′ UTR luciferase reporter construct was generated by cloning the CYLD mRNA 3′ UTR region into SacI/XmaI sites of pGL3 luciferase reporter plasmid (Promega, Madison, WI). The 3′ UTR regions of wild type and mutant CYLD were amplified by PCR using the following primers: CYLD-WT: 5′-CGAGCTCGAACT CCAGAGTTTCTTTGAAGGTTG-3′ (forward) and 5′-CCCGGGGACCATAAAAA CTGCATTTTAATGATAC-3′ (reverse). CYLD-Mut: 5′-CGAGCTCGCTCTGGCTTT AAACAAATTGC-3′ (forward) and 5′-CCCGGGGTGCAAAATGCTGCTTATTAA-3′ (reverse). The wild-type and mutant CYLD reporters were cotransfected with mock, lin4 mimics or miR-19a mimics, respectively. Forty-eight hours later, the cells were harvested and the luciferase activities were measured by a Dual-Luciferase Reporter Assay System Kit (Promega, USA). Firefly luciferase activity was normalized to Renilla luciferase activity. Three independent experiments were performed separately. The pGL3 was also utilized to produce miR-19a promoter luciferase reporter plasmid and the following primers were used for PCR amplification: miR-19a: 5′-CGAGCTCGCAAGCAATTTTCCTGCCTCA-3′ (forward) and 5′-CCCGGGGGTAGACACAGGTGTGGGCCCT-3′ (reverse).

### Real-time quantitative PCR (RT-qPCR)

Procedures for RT-qPCR analysis have been described previously [11, 36]. In brief, total RNA and miRNA were separately isolated by Trizol (Invitrogen) and by the mirVana miRNA Isolation Kit (Ambion). Complementary DNA for genes was synthesized with PrimeScript^®^RT Reagent Kit (Takana, Dalian, China) using Super Array PCR master mix (SuperArray Bioscience, USA) and cDNA for miR-19a was synthesized with Taqman miRNA Reverse Transcription Kit (Applied Biosystems, CA, USA). Real-time PCR was then performed on an Applied Biosystems 7900HT cycler using Takana SYBR^®^ Primix Ex Taq™Kit (Takana, China) or TaqMan miRNA Assay Kit (Applied Biosystems, USA) according to the manufacturer’s guidelines with the following primers: CYLD sense, 5′-CTCCTTTCCTGCGTCACACT-3′; CYLD antisense, 5′-TTTGATGGAGTGCAGCTTTG-3′; GAPDH sense, 5′-AATCCCATCACCATCTTCC-3′; GAPDH antisense, 5′-TGGACTCCACGACG TACTC-3′; miR-19a sense, 5′-CCTCTGTTAGTTTTGCATAGTTGC-3′ and miR-19a antisense, 5′-CAGGCCACCATCAGTTTTG-3′. RNU6b was employed as an internal control for miRNA and the levels of mRNA expression were defined based on Ct.

### Chromatin immunoprecipitation (ChIP) assay

ChIP assay was carried out using Pierce Agarose ChIP Kit (26156, Thermo) as described previously [11, 35]. In brief, cells (2×10^6^) plated in 100 mm culture dishes were treated with 1% formaldehyde to cross-link proteins to DNA. The cell lysates were sonicated to shear DNA to sizes of 300-1000 bp lengths. DNA-protein complexes were immunoprecipitated with 2 μg anti-RelA antibody or 2 μg anti-IgG antibody as a negative control. The immunoprecipitates were eluted and reverse crosslinked to purify the DNA fragments. Immunoprecipitated and input DNAs were subjected to RT-qPCR analysis. The specific primers for amplifying RelA-binding regions are as follows: VEGFA: 5′-TGCTCCTGGGGTGCTAGA-3′ (forward) and 5′-ACAATAAGAGTTAAGCAG-3′ (reverse); miR-19a: 5′-CTGGCTTCTCAGTG TGTTAT-3′ (forward) and 5′-CTGGAAATCTGACATGTAATC-3′ (reverse). The amount of precipitated DNA was calculated as the percentage of input sample and each sample was detected in triplicate.

### Cellular ubiquitination assay

Cellular ubiquitination assays were performed as previously described with some modifications [11, 35]. Briefly, cells transfected with the indicated plasmids were underwent various treatments prior to harvest. The cells were lysed in radio-immunoprecipitation assay (RIPA) buffer with protease inhibitors (KeyGene, China) and phosphatase inhibitor cocktail (KeyGene, China). His-tagged IκBα was eluted by incubating with Ni^2+^-nitrilotriacetic acid (NTA)-sepharose beads for 30 min at 4°C. Polyubiquitinated IκBα was detected using an anti-Ub antibody.

### Co-immunoprecipitation

For co-immunoprecipitation assays, cells were washed with 1×PBS and then solubilized on ice either in a radioimmunoprecipitation assay (RIPA) buffer (Cwbiotech, Beijing, China) containing 50 mM Tris [PH7.4], 150 mM NaCl, 1% NP-40, 0.25% sodium deoxycholate and protease inhibitors. After centrifugation at 14,000 rpm for 10 min at 4°C, the supernatants were transferred to the fresh tubes and then incubated with primary antibodies at 4°C followed by a further incubation with protein A/G-sepharose beads (Cwbiotech, Beijing, China) overnight. After rinsing three times with the lysis buffer, immunoprecipitated proteins were boiled for 10 min in sample buffer and analyzed by western blotting.

### Western-blotting

Western-blotting was performed with precast gradient gels (Bio-Rad) using standard methods as described previously [36, 37]. Briefly, total protein from each sample was resolved in 10% sodium dodecyl sulfate (SDS)-polyacrylimide gel electrophoresis and was transferred to the Immobilon™ PVDF Transfer Membranes (Millipore Corporation, Billerica, MA). The membrane was then blocked in 5% bovine serum albumin (BSA) and incubated with the primary antibodies against CYLD (1:1000, Cell Signaling Technology, USA), phospho-IKKβ (1:1000, Cell Signaling Technology), total IKKβ (1:1000, Cell Signaling Technology), total IκBα (1:1000, Cell Signaling Technology), RelA (1:1000, Cell Signaling Technology), Flag (1:1000; ProteinTech group, USA), proliferating cell nuclear antigen (PCNA) (1:2000, Biosynthesis, China) and GAPDH (1:3000, Biosynthesis). After incubation with HRP-linked secondary antibodies, the bands were visualized by western chemiluminscent HRP Substrate Kit (PPLYGEN, Beijing, China).

### Statistical analysis

SPSS software 17.0 (Chicago, IL, USA) was used for all statistical analyses. The unpaired, two-sided Student’s *t* test was used to assess comparisons between two groups. Comparisons among multiple groups were evaluated by one-way ANOVA. Data were expressed as mean ± standard deviation of at least three independent experiments and *P* value of < 0.05 was considered statistically significant.

## SUPPLEMENTARY MATERIALS FIGURES



## Abbreviations

CYLD: cylindromatosis
UTR: untranslated region
VEGF: vascular endothelial growth factor
TGF: tumor growth factor
β-TrCP: β-transducin repeat-containing protein
TAK1: TGFβ-activated kinase 1
DUB: deubiquitination
3′ UTR: 3′ untranslated region
