# Supplementary material for: Transcriptional downregulation of microRNA-19a by ROS production and NF-κB deactivation governs resistance to oxidative stress-initiated apoptosis
Source: Oncotarget. 2017 Aug 12;8(41):70967–81. doi: 10.18632/oncotarget.20235 (PMC5642611; doi:10.18632/oncotarget.20235)
Supplement: Supplementary file 1 [file oncotarget-08-70967-s001.pdf]

Transcriptional downregulation of microRNA-19a by ROS production and NF-κB deactivation governs resistance to oxidative stress-initiated apoptosis

SUPPLEMENTARY MATERIALS

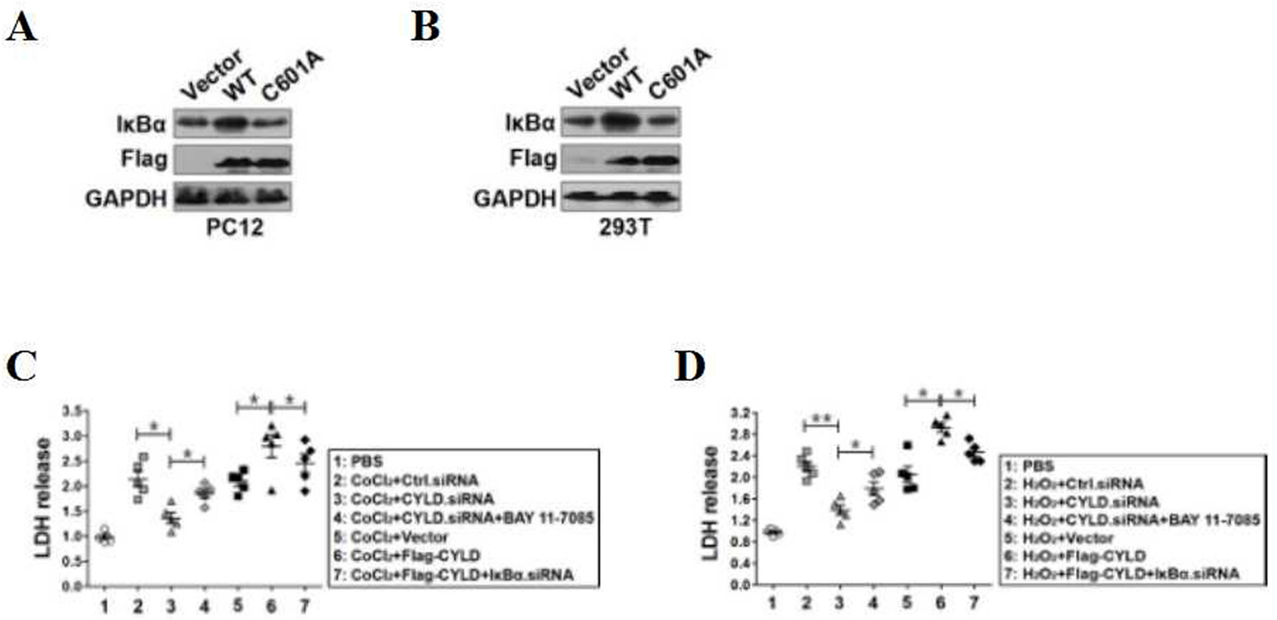

Supplementary Figure 1: CYLD enhances susceptibility to OS-initiated apoptosis by deactivating NF-κB. (A and **B)** Western-blotting examining abundance of IκBα protein in PC12 cells (A) and HEK 293T (B) cells transfected with vector, Flag-tagged wild-type CYLD (WT) and mutant C601A CYLD (C601A), respectively. **(C** and **D)** LDH release assay examining the levels of LDH release in PC12 cells exposed to 0.6 mmol/L $CoCl_2$ (C) or 0.4 mmol/L H_2O_2(D) with CYLD.siRNA transfection in the absence or presence of BAY 11-7085 administration and with Flag-CYLD transfection in the absence or presence of IκBα.siRNA transfection, respectively. Experiments were performed three times and data are expressed as mean ± s.d. **p* < 0.05; ***p* < 0.01, one-way ANOVA, post hoc comparisons, Tukey's test.

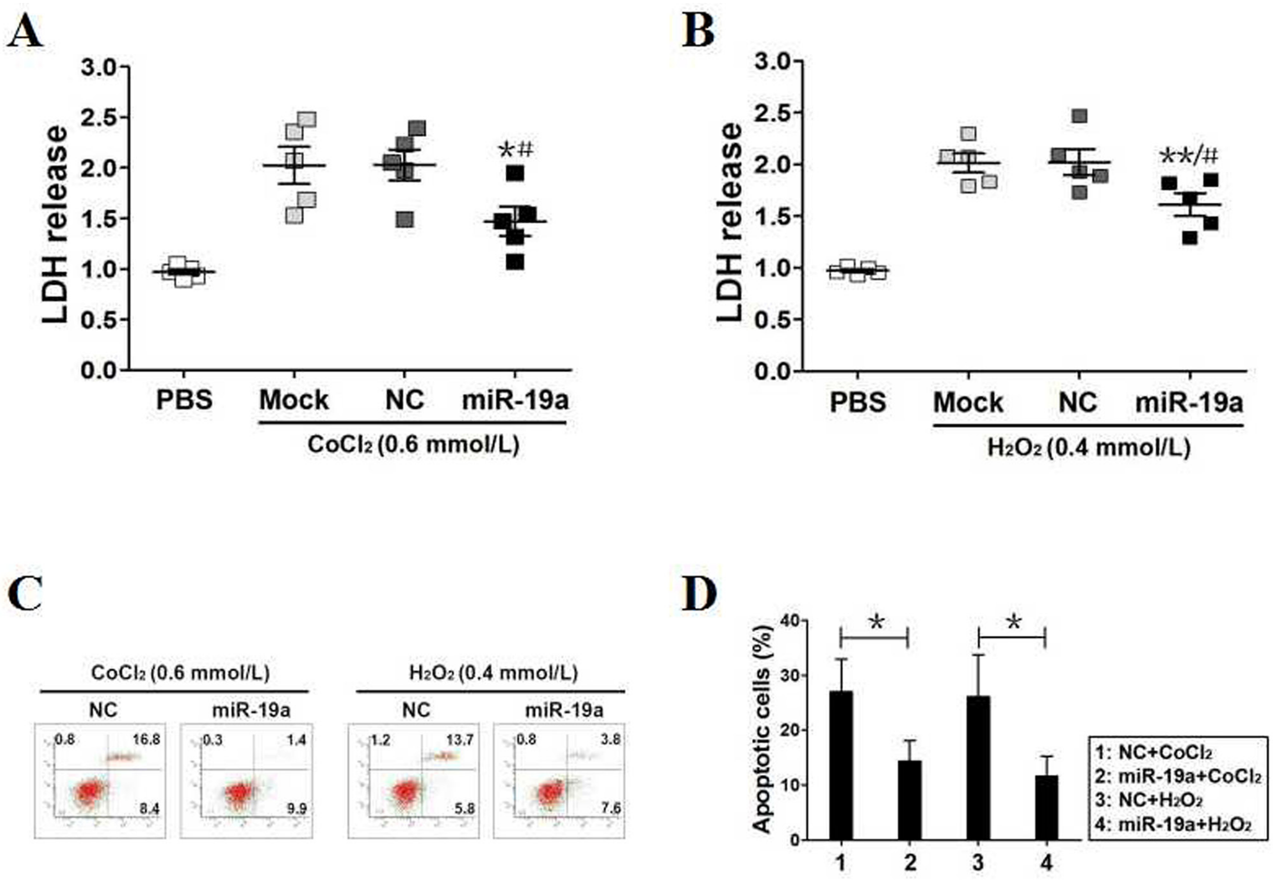

Supplementary Figure 2: MiR-19a antagonizes OS-initiated LDH release. (A) LDH release assay examining the levels of LDH release in PC12 cells exposed to 0.6 mmol/L $CoCl_2$ with mock (Mock), negative control (NC) and miR-19a mimics (miR-19a) transfection, respectively. Experiments were performed three times and data are expressed as mean ± s.d. **p* < 0.05 versus PBS; #*p* < 0.05 versus Mock and NC, one-way ANOVA, post hoc comparisons, Tukey's test. **(B)** LDH release assay examining the levels of LDH release in PC12 cells exposed to 0.4 mmol/L H_2O_2 with mock (Mock), negative control (NC) and miR-19a mimics (miR-19a) transfection, respectively. Experiments were performed three times and data are expressed as mean ± s.d. ***p* < 0.01 versus PBS; #*p* < 0.05 versus Mock and NC, one-way ANOVA, post hoc comparisons, Tukey's test. **(C** and **D)** Representative histograms (C) and quantification (D) of flow cytometry with Annexin-V/PI staining in PC12 cells exposed to 0.6 mmol/L $CoCl_2$ or 0.4 mmol/L H_2O_2 with negative control (NC) or miR-19a mimics transfection, respectively. Experiments were performed three times and data are expressed as mean ± s.d. **p* < 0.05, one-way ANOVA, post hoc comparisons, Tukey's test.

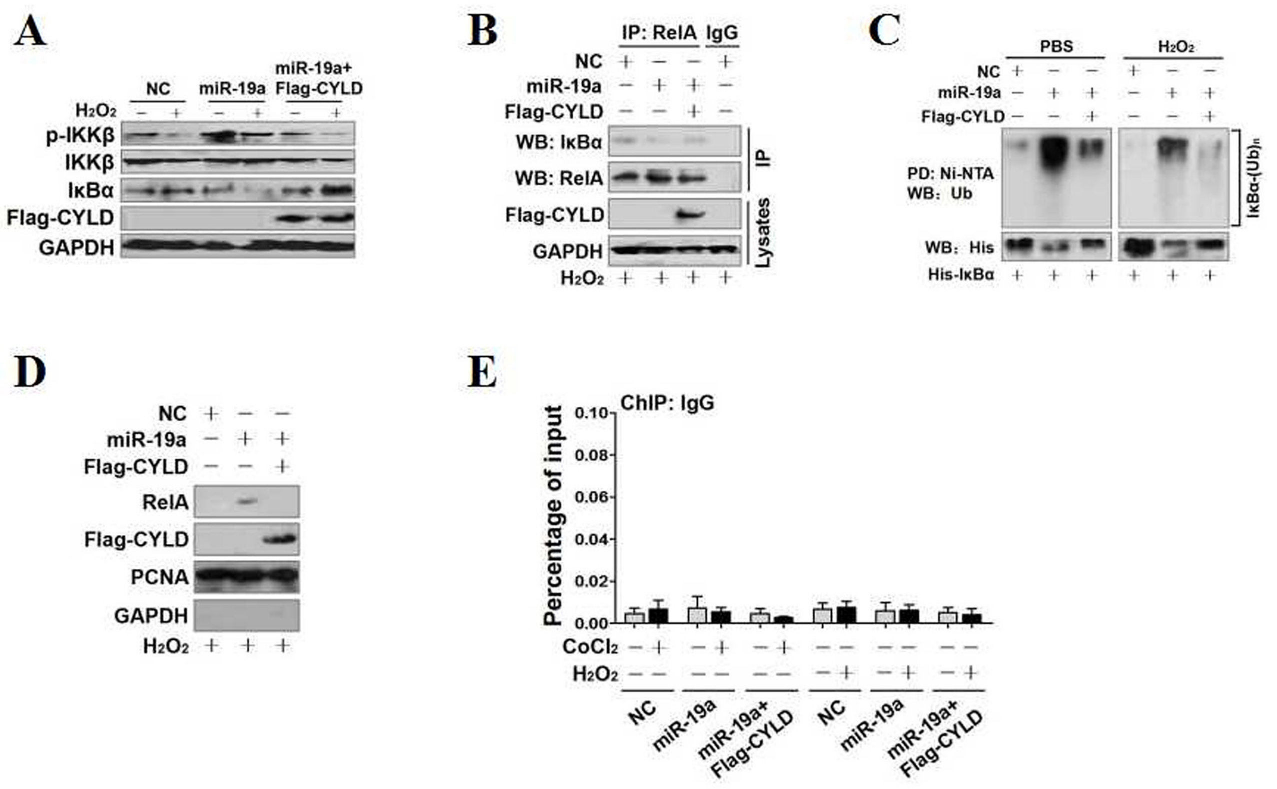

Supplementary Figure 3: CYLD repression is responsible for the NF-κB transactivation mediated by miRNA-19a during OS. **(A)** Western-blotting analyses comparing the levels of IKKβ phosphorylation and total IκBα expression in PC12 cells treated with 0.4 mmol/L H_2O_2 in the presence of miR-19a mimics transfection and miR-19a mimics plus Flag-tagged wild-type CYLD cotransfection, respectively. **(B)** Coimmunoprecipitation assays examining the interaction between RelA and IκBα in PC12 cells treated with 0.4 mmol/L H_2O_2 in the presence of miR-19a mimics transfection and miR-19a mimics plus Flag-tagged wild-type CYLD cotransfection, respectively. **(C)** Cellular ubiquitination assays comparing the poly-Ub levels of IκBα in PC12 cells treated with 0.4 mmol/L H_2O_2 in the presence of miR-19a mimics transfection and miR-19a mimics plus Flag-tagged wild-type CYLD cotransfection, respectively. **(D)** Western-blotting analyses detecting the levels of nuclear RelA accumulation in PC12 cells treated with 0.4 mmol/L H_2O_2 in the presence of miR-19a mimics transfection and miR-19a mimics plus Flag-tagged wild-type CYLD cotransfection, respectively. **(E)** ChIP analysis for IgG binding to *VEGFA* gene promoter in PC12 cells exposed to 0.6 mmol/L $CoCl_2$ and 0.4 mmol/L H_2O_2 in the presence or absence of miR-19a mimics transfection and miR-19a mimics plus Flag-tagged wild-type CYLD cotransfection, respectively. Enrichment of promoter region was normalized by input and data are expressed as mean ± s.d. of at least three experiments.

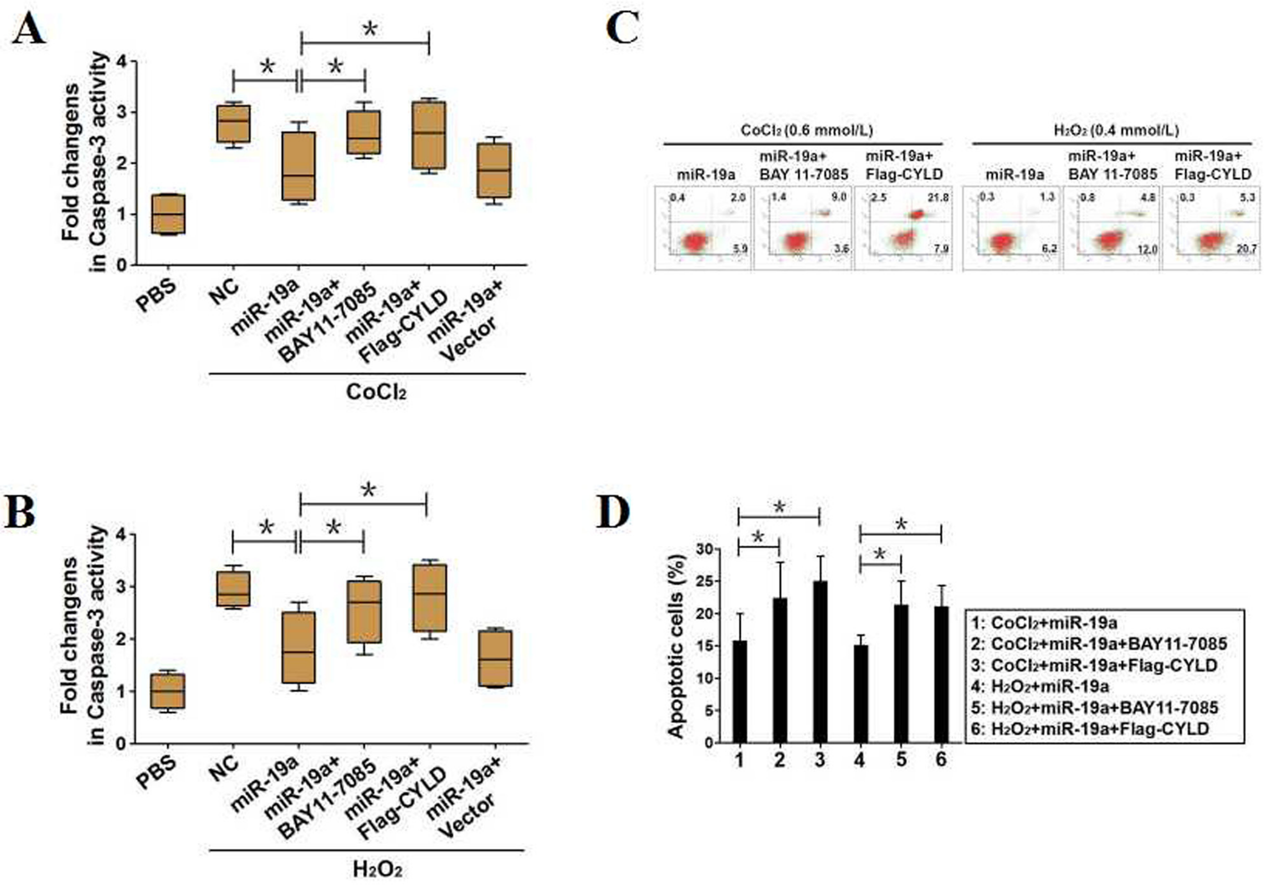

Supplementary Figure 4: CYLD repression and NF-κB transactivation promotes the resistant phenotypes of miR-19a in OS-initiated apoptosis. (A) Caspase-3 activity assays of PC12 cells exposed to 0.6 mmol/L $CoCl_2$ with miR-19a mimics transfection, with miR-19a mimics transfection plus 5 μmol/L BAY 11-7085 pretreatment, with miR-19a mimics plus Flag-tagged wild-type CYLD cotransfection and with miR-19a mimics plus empty vector cotransfection, respectively. Data are expressed as mean ± s.d. *$p < 0.05$, one-way ANOVA, post hoc comparisons, Tukey's test. **(B)** Caspase-3 activity assays of PC12 cells exposed to 0.4 mmol/L H_2O_2 with miR-19a mimics transfection, with miR-19a mimics transfection plus 5 μmol/L BAY 11-7085 pretreatment, with miR-19a mimics plus Flag-tagged wild-type CYLD cotransfection and with miR-19a mimics plus empty vector cotransfection, respectively. Data are expressed as mean ± s.d. *$p < 0.05$, one-way ANOVA, post hoc comparisons, Tukey's test. **(C and D)** Representative histograms (C) and quantification (D) of flow cytometry with Annexin-V/PI staining in PC12 cells exposed to 0.6 mmol/L $CoCl_2$ or 0.4 mmol/L H_2O_2 with miR-19a mimics transfection, with miR-19a mimics transfection plus 5 μmol/L BAY 11-7085 pretreatment, with miR-19a mimics plus Flag-tagged wild-type CYLD cotransfection, respectively. Experiments were performed three times and data are expressed as mean ± s.d. *$p < 0.05$, one-way ANOVA, post hoc comparisons, Tukey's test.

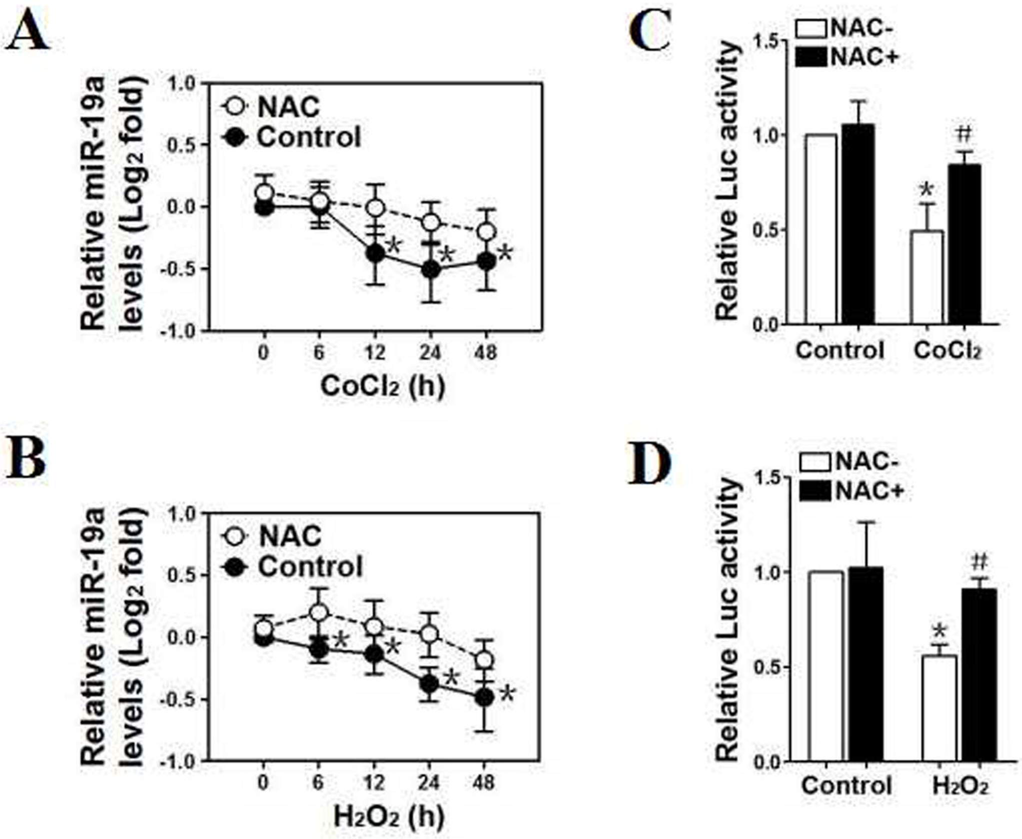

Supplementary Figure 5: OS impedes miR-19a transcription in a ROS-dependent manner. **(A)** RT-qPCR comparing levels of miR-19a mRNA expression in PC12 cells treated with $CoCl_2$ for the indicated times in the presence or absence of 2 mmol/L N-acetylcysteine (NAC) administration. Experiments were performed five times, each with quantitative RT-PCR in technical duplicate and real-time values were normalized to RNU6b. Data are expressed as mean ± s.d. $*p < 0.05$ versus NAC, one-way ANOVA, post hoc comparisons, Tukey's test. **(B)** RT-qPCR comparing levels of miR-19a mRNA expression in PC12 cells treated with H_2O_2 for the indicated times in the presence or absence of 2 mmol/L N-acetylcysteine (NAC) administration. Experiments were performed five times, each with quantitative RT-PCR in technical duplicate and real-time values were normalized to RNU6b. Data are expressed as mean ± s.d. $*p < 0.05$ versus NAC, one-way ANOVA, post hoc comparisons, Tukey's test. **(C)** Luciferase assays of miR-19a promoter activity in PC12 cells treated with $CoCl_2$ in the presence or absence of 2 mmol/L N-acetylcysteine (NAC) administration. Experiments were performed three times and data are expressed as mean ± s.d. $*p < 0.05$ versus control; $^{\#}p < 0.05$ versus $CoCl_2$, one-way ANOVA, post hoc comparisons, Tukey's test. **(D)** Luciferase assays of miR-19a promoter activity in PC12 cells treated with H_2O_2 in the presence or absence of 2 mmol/L N-acetylcysteine (NAC) administration. Experiments were performed three times and data are expressed as mean ± s.d. $*p < 0.05$ versus control; $^{\#}p < 0.05$ versus H_2O_2, one-way ANOVA, post hoc comparisons, Tukey's test.

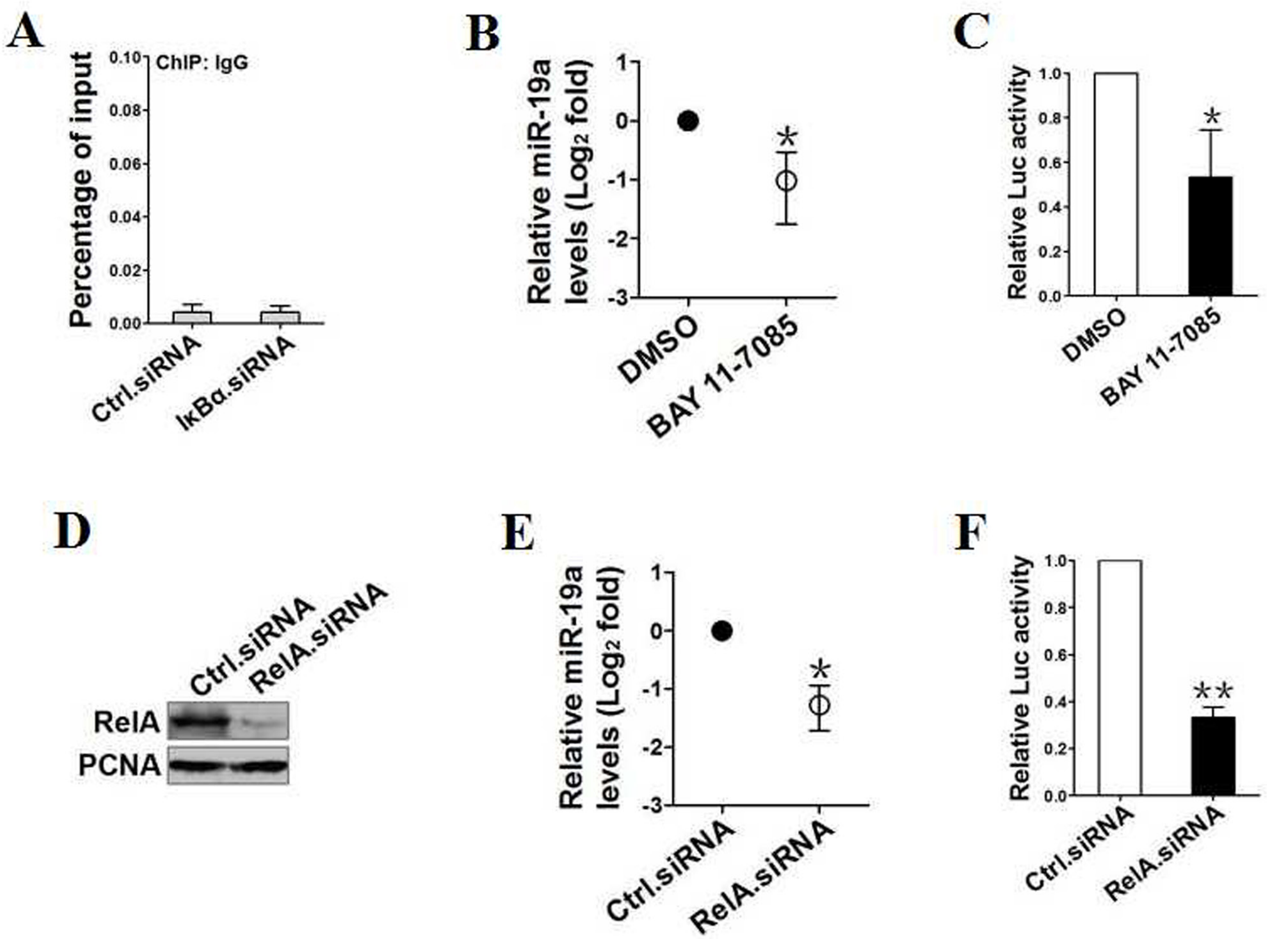

Supplementary Figure 6: Deactivation of NF-κB impedes miR-19a transcription. **(A)** ChIP analysis for IgG binding to miR-19a promoter in PC12 cells transfected with control.siRNA (Ctrl.siRNA) and IκBα.siRNA, respectively. Enrichment of promoter region was normalized by input and data are expressed as mean ± s.d. of at least three experiments. **(B)** RT-qPCR evaluating levels of miR-19a mRNA expression in PC12 cells treated with 5 μmol/L BAY 11-7085. Experiments were performed five times, each with quantitative RT-PCR in technical duplicate and real-time values were normalized to RNU6b. Data are expressed as mean ± s.d. $^*p < 0.05$. Two-sided Student's *t* test was used to calculate the *p* value. **(C)** Luciferase assays of miR-19a promoter activity in PC12 cells treated with 5 μmol/L BAY 11-7085. Experiments were performed three times and data are expressed as mean ± s.d. $^*p < 0.05$. Two-sided Student's *t* test was used to calculate the *p* value. **(D)** Western-blotting examining abundance of RelA protein expression in PC12 cells transfected with control.siRNA (Ctrl.siRNA) and RelA.siRNA, respectively. **(E)** RT-qPCR evaluating levels of miR-19a mRNA expression in PC12 cells transfected with control.siRNA (Ctrl.siRNA) and RelA.siRNA, respectively. Experiments were performed five times, each with quantitative RT-PCR in technical duplicate and real-time values were normalized to RNU6b. Data are expressed as mean ± s.d. $^*p < 0.05$. Two-sided Student's *t* test was used to calculate the *p* value. **(F)** Luciferase assays of miR-19a promoter activity in PC12 cells transfected with control.siRNA (Ctrl.siRNA) and RelA.siRNA, respectively. Experiments were performed three times and data are expressed as mean ± s.d. $^{**}p < 0.01$. Two-sided Student's *t* test was used to calculate the *p* value.

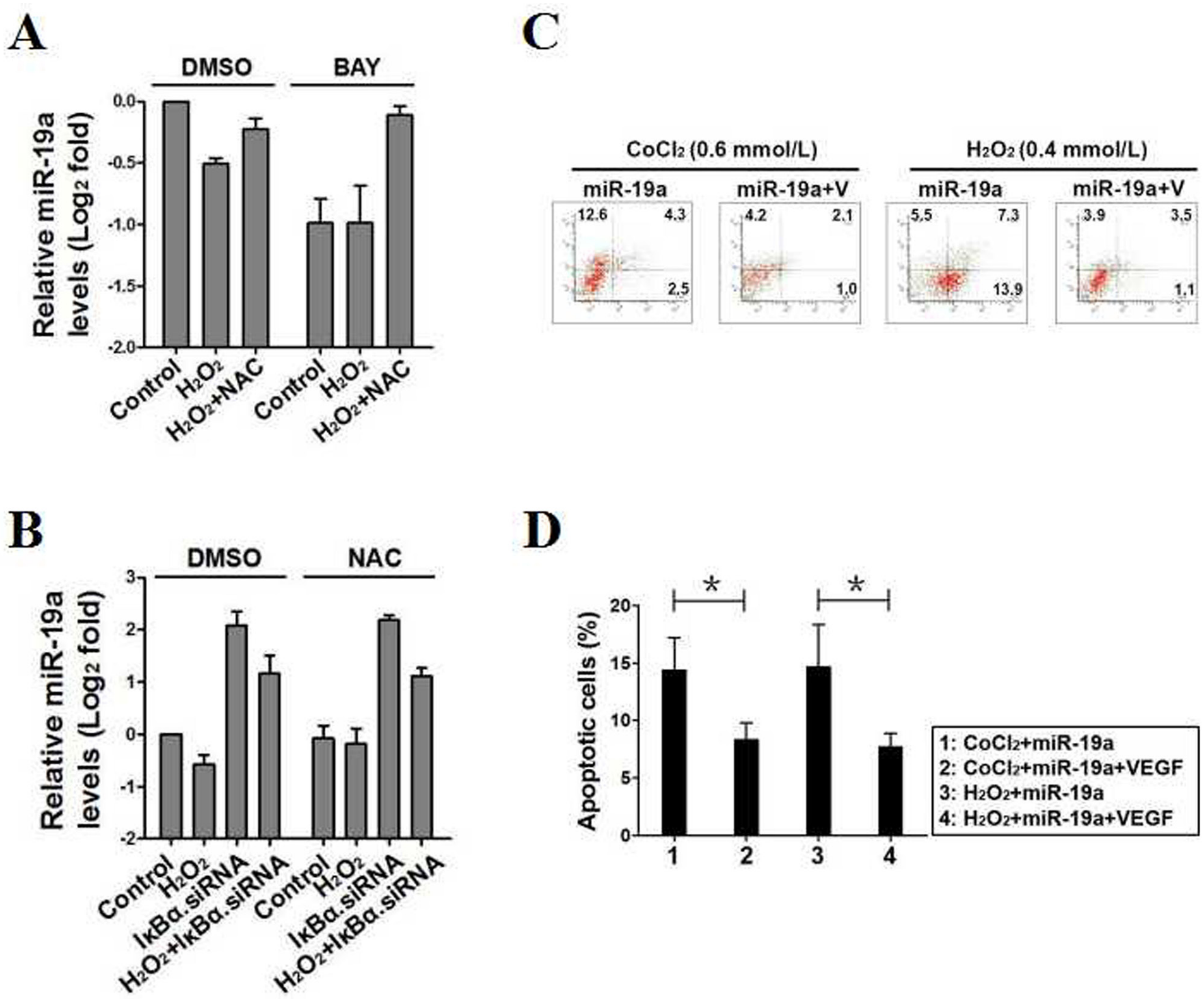

Supplementary Figure 7: MiR-19a is transcriptionally downregulated in response to OS through two distinct pathways orchestrated by ROS production and NF-κB deactivation. **(A)** RT-qPCR evaluating levels of miR-19a mRNA expression in H_2O_2-treated PC12 cells with N-acetylcysteine (NAC) treatment in the presence or absence of BAY 11-7085 administration. Experiments were performed five times, each with quantitative RT-PCR in technical duplicate and real-time values were normalized to RNU6b. Data are expressed as mean ± s.d. **(B)** RT-qPCR comparing levels of miR-19a mRNA expression in H_2O_2-treated PC12 cells with IκBα. siRNA transfection in the presence or absence of N-acetylcysteine (NAC) treatment. Experiments were performed five times, each with quantitative RT-PCR in technical duplicate and real-time values were normalized to RNU6b. Data are expressed as mean ± s.d. **(C and D)** Representative histograms (C) and quantification (D) of flow cytometry with Annexin-V/PI staining in PC12 cells exposed to 0.6 mmol/L $CoCl_2$ or 0.4 mmol/L H_2O_2 with miR-19a mimics transfection in the presence or absence of 100 ng/mL VEGF pretreatment. Experiments were performed three times and data are expressed as mean ± s.d. $*p < 0.05$, one-way ANOVA, post hoc comparisons, Tukey's test.
